# A belt-buckle checkpoint regulates the onset of botulinum neurotoxin intoxication

**DOI:** 10.1038/s41467-026-74499-7

**Published:** 2026-06-23

**Authors:** Baohua Chen, Linfeng Gao, Melvin Bönninger, Ting Huang, Nadja Krez, Weihua Wen, Mark Bowen, Jianlong Lou, James D. Marks, Andreas Rummel, Rongsheng Jin

**Affiliations:** 1 Department of Physiology and Biophysics, University of California, Irvine, Irvine, CA USA; 2 Institute of Toxicology, Hannover Medical School, Hannover, Germany; 3 Department of Anesthesia, University of California, San Francisco, Zuckerberg San Francisco General Hospital, San Francisco, CA USA; 4 Department of Physiology & Biophysics, Stony Brook University, Stony Brook, NY USA

**Keywords:** Cryoelectron microscopy, Pathogens, Infectious diseases, Recombinant protein therapy

## Abstract

Fast-acting botulinum neurotoxins (BoNTs) are highly desirable for both medical and aesthetic indications, but the underlying mechanism for the differing onset of BoNTs’ action remains unknown. Here, we demonstrate that the “belt” of BoNTs, a largely unstructured loop wrapping around their catalytic light chain (LC), is key to onset of intoxication. The more flexible BoNT/E belt promotes quicker LC translocation into the neuronal cytosol, leading to faster onset of action compared to BoNT/A. Furthermore, we discover a “belt-buckle” checkpoint that regulates this process. By loosening the BoNT/A belt-buckle via protein engineering, we enhance its sensitivity to acidic pH, leading to an accelerated onset of action. Conversely, locking the belt-buckle with an antibody neutralizes BoNT/A. Our findings open avenues for developing fast-acting BoNTs and effective countermeasures.

## Abstract (web summary)

Here the authors perform structural studies showing a molecular “belt-buckle” checkpoint that regulates the speed of botulinum neurotoxin action, revealing strategies for both therapeutic toxin engineering and toxin neutralization.

## Introduction

Botulinum neurotoxins (BoNTs) comprise seven established serotypes (termed BoNT/A–G)^1^. However, BoNT/A and BoNT/B are the only two to have received FDA approval for medical and aesthetic applications so far, following research spanning over half a century. Currently, almost all commercial BoNT products worldwide are based on BoNT/A, which exhibits a high potency and a long duration of action. In comparison, the only available BoNT/B suffers from low potency in humans due to a genetic variation in its protein receptor synaptotagmin 2^2,3^. In addition, positive topline results from Phase 3 clinical studies evaluating BoNT/E have been reported recently^4,5^. Interestingly, BoNT/E takes clinical effect usually within one-day post-injection, which is much faster than that of BoNT/A (6-7 days) and BoNT/B^4–11^. However, BoNT/E exhibits lower potency and a much shorter duration of action compared to BoNT/A. This underscores the pressing need to accelerate the onset of action for potent, long-lasting BoNTs, such as BoNT/A, which would be highly beneficial for treating neurologic conditions requiring rapid intervention and timely outcome assessment, as well as for aesthetic applications where faster results are desired. However, the molecular mechanisms that dictate the varying speed of effect onset among various BoNTs remain largely unknown.

**Fig. 1:**
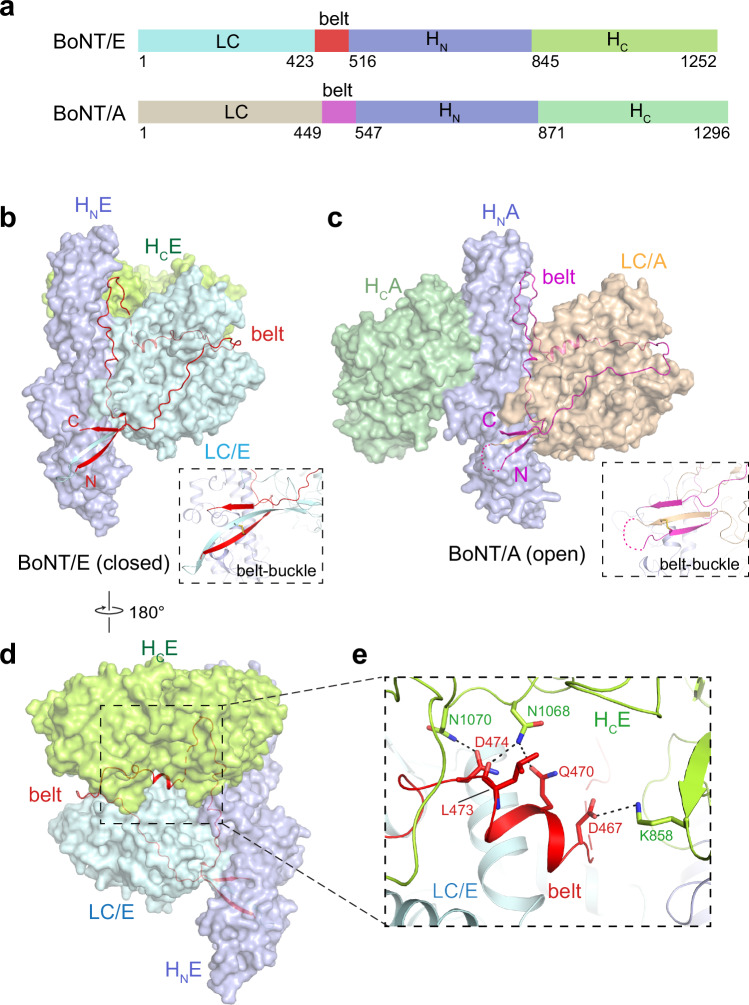
Structural comparison of the belts of BoNT/E and BoNT/A in the context of holotoxins. **a** Schematic representation of the domain organizations of BoNT/E and BoNT/A. **b**, **d** The crystal structure of BoNT/E (PDB: 3FFZ) is shown in surface (**b**) and cartoon (**d**) representations, which are related by a 180° rotation around a vertical axis. The belt is highlighted in red. **c** The crystal structure of BoNT/A (PDB: 3BTA) is shown in a surface representation, with its belt colored in magenta. The belt-buckle areas, where the N- and C-termini of the belt associate with the C-terminal tail of the LC, are highlighted in dashed boxes. **e** A close-up view of the interface between the BoNT/E belt and H_C_E, with the belt shown in red, H_C_E in lime green, and LC/E in pale cyan. The key interacting residues are shown as sticks with hydrogen bond and salt bridge interactions shown as dashed lines.

Each BoNT molecule is composed of a ~50 kDa light chain (LC) and a ~100 kDa heavy chain (HC), where the HC comprises an N-terminal translocation domain (H_N_) and a C-terminal receptor-binding domain (H_C_) (Fig. 1a)^1^. BoNTs specifically target cholinergic neurons through their H_C_ domains that synergistically bind complex gangliosides and specific protein receptors on the neuron surface^12–15^. Upon entering neurons by receptor-mediated endocytosis and triggered by vesicle acidification, the LC partially unfolds and translocates across the endosomal membrane, facilitated by the H_N_. The LC then refolds in the cytosol and specifically cleaves SNARE (soluble N-ethylmaleimide-sensitive factor attachment protein receptor) complexes. This blocks vesicle fusion and prevents the release of acetylcholine, resulting in paralysis of the affected muscles^1,16–18^. Both BoNT/A and BoNT/E exploit synaptic vesicle glycoprotein 2 (SV2) as the receptor^19–25^ and cleave the same substrate SNAP-25 with similar activity^9^, suggesting that receptor binding and LC activity are unlikely to be the major determinants for the significantly different speed of onset of action between BoNT/A and BoNT/E.

The crystal structures of BoNT/A and BoNT/B exhibit a linear “open-wing”-like arrangement at neutral pH where the H_C_ and LC are located on opposite sides of the long helical H_N_, while a recent cryogenic electron microscopy (cryo-EM) study revealed that BoNT/A also exhibits a semi-closed conformation in solution^26–29^. In contrast, the H_C_ and the LC-H_N_ of BoNT/E fold toward each other, resulting in a “closed-wing” conformation at neutral pH that was observed in both the crystal and cryo-EM structures (Fig. 1b, c)^29,30^. It was speculated that the closed-wing conformation of BoNT/E may be more efficient for LC translocation, and BoNT/A and B may switch into the BoNT/E-like closed-wing conformation prior to acidic-pH-triggered LC unfolding and translocation^9,30^. However, how BoNT/E responds to endosome acidification remains unknown.

Here, we investigate the structure of BoNT/E at acidic pH, mimicking the endosomal environment, using cryo-EM. Surprisingly, we find that BoNT/E adopts an ensemble of open conformations at acidic pH with its H_C_ moving away from the LC-H_N_ domain. Notably, we find that the N-terminal region of the HC of BoNT/E (residues K423–N515), which is largely unstructured and wraps around the LC via extensive interactions (hence referred to as the “belt” segment), exhibits greater flexibility compared to the belts of BoNT/A and BoNT/B. Prior studies suggested that the belt is a surrogate pseudo-substrate inhibitor of the LC protease, which embraces the LC and stabilizes its compacted conformation^31^. These findings suggest that the looser belt of BoNT/E, compared to BoNT/A, may exert a weaker constraint on LC/E, allowing it to unfold more rapidly. This could lead to quicker translocation and earlier action of LC/E in the cytosol, ultimately resulting in a faster onset of clinical symptoms.

To explore this hypothesis, we performed reverse engineering on the slower-acting BoNT/A and examined whether loosening its belt by structure-based mutagenesis could accelerate its onset of action. These experiments lead us to identify a functional hotspot on the belt, where its N- and C-termini, after wrapping around the LC like a strap, flank the C-terminal tip of LC to form a buckle-like motif (termed belt-buckle). We find that destabilizing the belt-buckle of BoNT/A by mutagenesis sped up its acidic pH-triggered conformational changes. An engineered BoNT/A holotoxin comprising the destabilized belt-buckle exhibited an accelerated speed of onset. Remarkably, we find that a human monoclonal antibody (2G11)^32–35^, which is one component of an antitoxin currently in clinical trials, potentially neutralizes BoNT/A by stabilizing the belt-buckle area. Taken together, these studies demonstrate that the belt-buckle of BoNT serves as a critical speed-limiting checkpoint during its action, opening avenues for developing fast-acting BoNTs while also laying the groundwork for further exploration of BoNT pathogenesis and potential countermeasures.

## Results

### The belt of BoNT/E is more flexible than that of BoNT/A and BoNT/B

BoNTs are initially synthesized as single polypeptide chains, which are cleaved by proteases at a surface-exposed linker connecting the LC and HC to yield the mature, biologically active toxins. The belt is a long, largely unstructured loop corresponding to the N-terminal segment of the HC immediately downstream of the cleavage site. After cleavage, the N-terminus of the belt remains covalently linked to the C-terminal end of the LC through a disulfide bond. The first segment of the BoNT/E belt, consisting of residues K423–D456, extends in parallel to the long helices of H_N_E and interacts with both LC/E and H_N_E. The second segment of the belt, consisting of residues T457–N515, fully encircles LC/E much like a strap and loops back to the starting point of the belt (Fig. 1b, d). Notably, the C-terminal tip of LC/E, forming a β-strand, is sandwiched between the N- and C-termini of the belt with each forming a β-strand, resembling a strap passing through a buckle (termed belt-buckle) (Fig. 1b, inset).

Structural comparison showed that the belt of BoNT/E does not interact with its LC-H_N_ fragment as tightly as that of BoNT/A and BoNT/B. For example, while the complete belts of BoNT/A and BoNT/B are well-defined in their structures due to extensive interactions between the belt and the LC, residues P499–N515 and N461–E465 of the BoNT/E belt are too flexible to be resolved in the cryo-EM and crystal structures, respectively, indicating weak or no interactions with LC/E (Supplementary Fig. 1a)^29,30^. Another distinct feature of the BoNT/E belt is that a segment spanning residues T457–N476 is sandwiched between LC/E and H_C_E in the context of its closed-wing conformation, burying ~404 Å^2^ of solvent accessible interface between the belt and H_C_E (Fig. 1d, e)^29,30^. These additional supports from H_C_E are crucial for holding the belt in place, as evidenced by the observation that, in the absence of H_C_E, almost the entire second segment of the BoNT/E belt (residues T459–A496) is too flexible to be resolved in the crystal structure of LC-H_N_/E (Supplementary Fig. 1b)^36^. In contrast, the belts of BoNT/A and BoNT/B do not interact with their H_C_, and they wrap around their respective LC tightly with fully visible electron densities in the crystal structures of LC-H_N_/A and LC-H_N_/B in the absence of their H_C_ domains^37,38^. These findings suggest that the belt of BoNT/E is intrinsically more flexible and associates less tightly with its LC-H_N_ fragment than that of BoNT/A and BoNT/B, requiring additional support from H_C_E in the context of the distinct closed-wing conformation.

### The belt of BoNT/E turns even more flexible at acidic pH

**Fig. 2:**
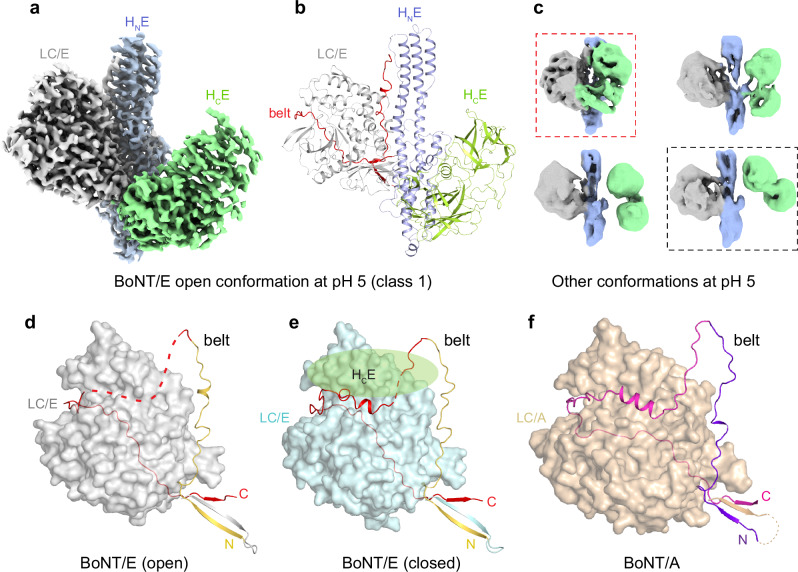
BoNT/E adopts various open conformations with a flexible belt at acidic pH. **a**, **b** Cryo-EM structure of BoNT/Ei in the M-PTC conformation (Class 1) at pH 5. LC/E is shown in light gray, the belt in red, H_N_E in light blue, and H_C_E in lime green. **c** Cryo-EM maps of BoNT/Ei in four minor classes at pH 5, revealing multiple open conformational states. The BoNT/A-like semi-closed and open conformations are outlined in a red or black dashed box, respectively. The color scheme follows that of (**a**). **d**–**f** Structural comparison of the belts among the open and closed conformations of BoNT/E, as well as BoNT/A. The dashed lines indicate areas with unresolved structures. H_C_E in the closed conformation of BoNT/E that helps hold the belt is shown as a green oval for clarity in (**e**).

The distinct closed-wing conformation of BoNT/E observed at neutral pH and the greater flexibility of its belt prompted us to ask how BoNT/E, especially its belt, responds to acidic pH, a signal that triggers LC unfolding and membrane translocation once inside synaptic vesicles. To this end, we determined the cryo-EM structure of BoNT/Ei, a genetically inactivated form, at pH 5 (Supplementary Fig. 2 and Supplementary Table 1). Surprisingly, analysis of the cryo-EM data yielded a series of EM maps revealing an ensemble of open conformations of BoNT/E, in which the LC-H_N_ moiety remains largely unchanged while H_C_E adopts various orientations (Fig. 2a–c and Supplementary Fig. 2). We determined the structure of a major conformation (Class 1) of BoNT/E holotoxin at 3 Å resolution, using ~64.46% of the total particles (Fig. 2a, b and Supplementary Fig. 2). We also reconstructed low-resolution maps for four minor classes of BoNT/E, using ~23.68%, ~6.51%, ~2.85%, and ~2.50% of the total particles, respectively (Fig. 2c and Supplementary Fig. 2). Interestingly, we observed a BoNT/A-like open-wing conformation and a semi-closed conformation that resembles the conformations of BoNT/A and tetanus neurotoxin (TeNT) observed in cryo-EM structures^26,39^ within these minor classes (Fig. 2c). These findings suggest that BoNT/E transitions from the closed-wing conformation to an ensemble of open states in response to vesicle acidification, with its H_C_ moving away from the largely rigid LC-H_N_ fragment.

Notably, such re-orientation of H_C_E would abolish its interactions with the belt and LC/E, a key feature of the closed-wing conformation of BoNT/E at neutral pH. Consistent with this finding, most of the second segment of BoNT/E belt (T457–K487) becomes too flexible to be resolved in the Class 1 conformation (Supplementary Fig. 1d), resembling the structure of the isolated LC-H_N_/E but not its conformation in the closed-wing state at neutral pH (Fig. 2d, e)^29,30^. We further did a focused refinement targeting the LC-H_N_ fragment and resolved another structure at 2.99 Å resolution, which confirmed that this segment of the BoNT/E belt is too flexible to be resolved (Supplementary Figs. 1e, 2, and 3a, b). These findings suggest that the flexibility of the BoNT/E belt significantly increases upon vesicle acidification, partly due to the loss of support from H_C_E as it moves away from LC-H_N_/E, which would reduce the belt constraints on LC/E, facilitating LC unfolding and transit into the next functional state.

Interestingly, the major conformation of BoNT/E at pH 5 (Class 1) resembles that of BoNT/E and BoNT/A when they are bound with another clostridial protein, non-toxic non-hemagglutinin (NTNH), at acidic pH (Supplementary Fig. 3c)^40–42^. NTNH is one of the neurotoxin-associated proteins (NAPs) encoded in the same gene cluster as the corresponding BoNT, which assembles with BoNT into a highly stable minimal progenitor toxin complex (M-PTC). The M-PTC provides both proteins with high stability against acidic pH and digestive proteases in the gastrointestinal (GI) tract during oral intoxication^40,41,43^. Exhibiting a spectrum of open conformations at acidic pH, particularly the Class 1 conformation, BoNT/E could readily assemble with NTNH/E. Within the M-PTC, a portion of the BoNT/E belt is protected by the nH_C_ domain of NTNH/E, resembling the stabilizing support from H_C_E in the closed-wing conformation^41^. Therefore, the increased vulnerability of BoNT/E in the open conformation to degradation in the acidic segments of the GI tract—particularly its more flexible belt—is mitigated by NTNH/E in the M-PTC.

### Enhancing the flexibility of the belt of BoNT/A by protein engineering

The belt plays important roles in the unfolding of the LC and reorganization of the H_N_ to form a protein-conducting channel^31,44^. Here, our cryo-EM studies reveal that the belt of BoNT/E is more flexible than that of BoNT/A and BoNT/B, and its flexibility increases at acidic pH. This distinct feature of the BoNT/E belt may help reduce the activation energy for acidic pH-induced LC unfolding and translocation across the H_N_ channel, ultimately leading to a fast onset of BoNT/E action. To validate this hypothesis, one could attempt to strengthen the interactions between the BoNT/E belt and the rest of the toxin through protein engineering, with the expectation of slowing down the action of BoNT/E. However, this approach is technically challenging due to its susceptibility to potential artifacts arising from non-specific interfering effects of mutagenesis. To address this challenge, we took a reverse engineering approach by using the slower-acting BoNT/A as a model and investigating whether loosening its belt through structure-based mutagenesis could promote a faster onset of action.

**Fig. 3:**
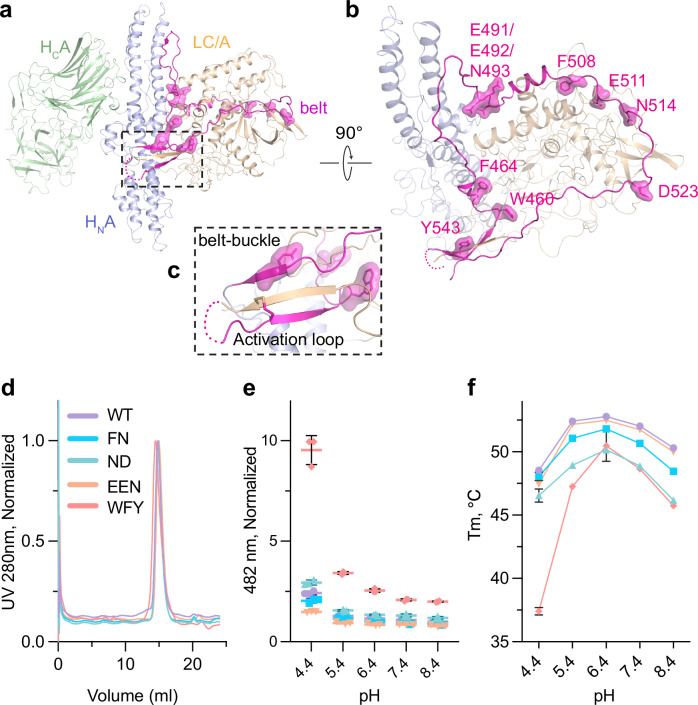
Mapping functional hotspots on the belt of BoNT/A that regulate its constraints on LC/A. **a**, **b** Crystal structure of BoNT/A (PDB: 3BTA) in cartoon representation. LC/A is colored wheat, H_N_A in light blue, H_C_A in pale green, and the belt in magenta. The selected LC/A-interacting residues on the belt that were subjected to mutagenesis studies are shown as sticks and surfaces. **c** A close-up view of the belt-buckle on BoNT/A. **d** The elution profiles of gel filtration chromatography of four LC-H_N_/A mutants compared to the WT protein. **e** pH-dependent unfolding of LC-H_N_/A variants probed by ANS dye. The values of fluorescence intensity at 482 nm were normalized to that of WT LC-H_N_/A at pH 8.4. **f** Thermal stability of LC-H_N_/A variants at the indicated pH. The data in (**e**, **f**) are presented as mean ± SD; *n* = 3.

Our strategy was to rationally mutate key amino acids on the belt that anchor it to LC/A, while avoiding alterations to other parts of the toxin. Guided by the crystal structure of BoNT/A, we identified an extensive protein-protein interaction network between the belt and LC/A that mutually stabilizes each other. We designed five variants of BoNT/A with targeted mutations across distinct regions of the belt, aiming to identify hotspot residues that mediate key interactions and to effectively disrupt the belt–LC/A association without impairing the function of LC/A (Fig. 3a–c). One variant contains mutations of three amino acids (E491G/E492G/N493G, referred to as EEN), which are located at the start of the second segment of the belt. Three variants involve mutations of four amino acids that are located in the middle section of the belt: F508G/E511G/N514G/D523G (termed FEND); F508G/N514G (FN); and N514G/D523G (ND). Another variant carries mutations of three amino acids, W460G/F464G/Y543G (WFY), which are located in the belt-buckle where the N- and C-termini of the belt associate with the C-terminal tail of the LC. Disrupting the belt–LC/A association by such designs would increase the flexibility of the belt, and thus reduce its constraint on LC/A, without affecting the enzymatic activity of LC/A or the overall structural integrity of the toxin.

We used the recombinant LC-H_N_/A (M1–N875) as a template to characterize these belt mutations. The behavior of these LC-H_N_/A variants in response to acidification should closely resemble that of the full-length BoNT/A because H_C_A neither interacts with the belt nor undergoes pH-dependent unfolding by itself. We found that the EEN, FN, ND, and WFY mutants of LC-H_N_/A could be successfully expressed in *E. coli* and purified to homogeneity in a manner comparable to the wild-type LC-H_N_/A, and they were eluted as WT-like single peaks on a size-exclusion chromatography (SEC) (Fig. 3d). These results demonstrate that mutations introduced in the belt of these four LC-H_N_/A variants do not affect proper protein folding or their overall structural integrity. In contrast, the FEND mutant showed low expression and poor purity, indicating compromised protein folding, and was not pursued for further studies.

### The belt-buckle regulates BoNT/A’s susceptibility to acidic pH-triggered conformational changes

We exploited two independent assays to examine how the various mutations introduced into the belt might affect the pH-dependent conformational changes of LC-H_N_/A. First, we used the environment-sensitive dye 8-anilinonaphthalene-1-sulfonic acid (ANS) as an indicator to examine the structural integrity of LC-H_N_/A at various pH values. ANS dye displays increased fluorescence intensity upon binding to hydrophobic regions of a protein, which become progressively more exposed during unfolding. We found that the ANS fluorescence intensity of the WT LC-H_N_/A increased when the pH decreased from 8.4 to 4.4, indicating that it gradually unfolded during acidification (Fig. 3e). The behaviors of the FN, ND, and EEN mutants were similar to that of the WT protein, suggesting the mutations they carried in the belt did not have a detectable effect on pH-dependent unfolding of LC-H_N_/A. However, the behavior of the WFY mutant was drastically different from that of others. For example, at neutral pH, the WFY mutant already showed a slightly elevated ANS intensity compared to the WT protein. Upon acidification, the increase in ANS intensity occurred more quickly and to a greater extent for the WFY mutant than the WT, reaching ~4-fold differences at pH 4.4 (Fig. 3e). Therefore, the WFY mutant is more sensitive to acidic pH, unfolding more rapidly and extensively than the WT protein under these conditions. These findings suggest that the stabilizing effect of the belt on the LC and H_N_ is not uniformly distributed across it, with the belt-buckle playing a pivotal role in this process. To verify proper folding of the WFY mutant, we conducted thrombin cleavage on both the WT and the WFY mutant, each containing a thrombin cleavage site inserted between LC/A and H_N_/A (Supplementary Fig. 4a). The cleaved LC/A and H_N_/A migrated separately under the reducing conditions. Under the non-reducing condition, however, the two fragments of the WFY mutant co-migrated as a WT-like single species, demonstrating that the connecting disulfide bond formed correctly.

We conducted a fluorescence-based thermal shift assay to further examine how these mutations affect the pH-dependent unfolding of LC-H_N_/A (Fig. 3f). This assay assesses the thermal stability of a protein, monitoring changes in fluorescence of an environmentally sensitive dye as the protein unfolds with increasing temperature. A change in the temperature (T_m_) indicates alterations in protein stability. We examined the T_m_ of these LC-H_N_/A variants at pH ranging from 8.4 to 4.4. Compared to the WT LC-H_N_/A, the FN, ND, and EEN mutants showed similar or slightly decreased T_m_ values at each tested pH, while the WFY mutant exhibited the largest changes. Notably, the WFY mutant exhibited a drastic 11 °C drop of its T_m_ at pH 4.4 compared to the WT protein, indicating that the WFY mutations in the belt-buckle significantly destabilized LC-H_N_/A at acidic pH. These results are consistent with the ANS studies, collectively highlighting the belt-buckle as a critical regulating checkpoint for stabilizing the LC and H_N_, while loosening the belt facilitates the acidic pH-triggered conformational change of LC-H_N_/A.

### BoNT/A-WFY displays a faster onset of action on neurons compared to the WT BoNT/A

**Fig. 4:**
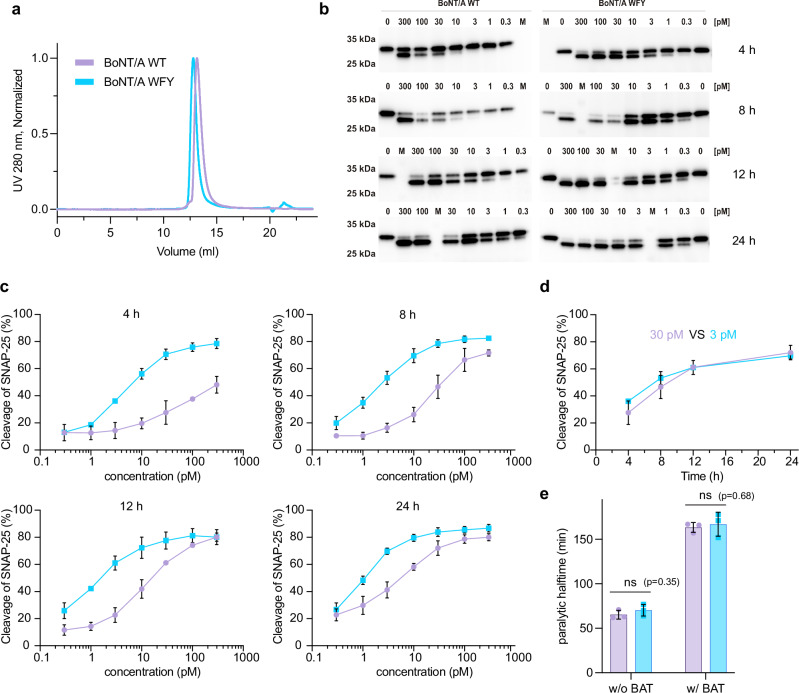
Functional characterization of the engineered BoNT/A-WFY. **a** The WT BoNT/A and BoNT/A-WFY are monodispersed in solution based on SEC analysis. **b**, **c** Mouse P19 neurons (P19N) were treated with WT BoNT/A or BoNT/A-WFY at indicated concentrations, washed after 2 h, and subsequently subjected to western blot analyses using a monoclonal anti-SNAP-25 antibody at 4, 8, 12, and 24 h post BoNT addition. Synaptobrevin2 was used as a loading control (Supplementary Fig. 4d). Representative immunoblots from one of three independent experiments are shown in (**b**). The percentage of SNAP-25 cleavage by the WT BoNT/A or BoNT/A-WFY was quantified using ImageJ and analyzed by GraphPad Prism 10 software (**c**). **d** Comparing the activity of 3 pM of BoNT/A-WFY and 30 pM of WT BoNT/A at 4–24 h. **e** Dosages of WT BoNT/A (3.3 pM) and BoNT/A-WFY (7.5 pM) causing similar paralytic halftimes were pre-incubated with 3.5 mU/mL of BAT^®^ or without antitoxin for 60 min at 37 °C and then tested in the MPN-BNA assay to quantify neutralization by this licensed equine polyclonal botulism antitoxin. Error bars indicate mean ± s.d., *N* = 3 (biologically independent experiments); ns, not significant (unpaired Student’s *t* test, two-sided).

We next examined whether weakening the belt-buckle with the WFY mutations might affect the structure and function of BoNT/A. To this end, we recombinantly produced the wild-type and WFY variant of BoNT/A holotoxin in *E. coli*. Each construct contains a thrombin recognition site engineered into the flexible linker connecting LC and H_N_, enabling controlled activation without disturbing the structure or activity of BoNT/A^23,44,45^. BoNT/A-WFY could be purified to homogeneity and activated in a manner comparable to WT BoNT/A (Fig. 4a and Supplementary Fig. 4b). We also produced the inactivated BoNT/Ai-WFY for structural characterization.

To assess whether weakening the belt affects the overall architecture of BoNT/A, we performed negative-stain EM on BoNT/Ai-WT and BoNT/Ai-WFY, followed by 2D classification. Representative class averages from both samples showed similar particle shapes and domain organization, broadly resembling the canonical structure of wild-type BoNT/A (PDB: 3BTA)^27^. In these averages, the LC, H_N_, and H_C_ domains could be readily recognized based on the reference structure (Supplementary Fig. 5a, b). While some structural differences may exist in BoNT/Ai-WFY, the gross features of the particles appear to be preserved at the negative-stain level. Our attempts to determine a high-resolution structure of BoNT/Ai-WFY by cryo-EM were unsuccessful, and only a low-resolution density map was obtained (Supplementary Fig. 6a, b). These findings are consistent with increased structural flexibility, likely arising from loosening of the belt region.

We then investigated the biological activity of BoNT/A-WFY at the neuromuscular junctions using the mouse phrenic nerve hemidiaphragm (MPN) assay^46^. This well-accepted ex vivo method examines the biological activity of BoNT by recording the contractions of an indirectly stimulated mouse hemidiaphragm mounted in an organ bath in the presence of BoNT at picomolar concentrations. The time interval between the administration of BoNT and the point when the muscle contraction amplitude is halved to its original value (paralytic halftime or *t*_1/2_) serves as the readout of toxin potency. We found that BoNT/A-WFY is highly potent, still exhibiting ~37% biological activity of the WT BoNT/A (Supplementary Fig. 4c).

In the MPN assay, the hemidiaphragm paralysis evoked by BoNT is detected within 2–3 h, which comprises indirect electrical stimulation leading to neurotransmitter release, followed by depolarization and contraction of muscle tissue. The essential indirect stimulation also causes accelerated uptake of BoNT into the motor neurons^47,48^. To carefully dissect the onset of BoNT activity, the direct measurement of intracellular SNAP-25 cleavage in non-stimulated conditions over a period of up to 24 h was performed in a cell-based neuron assay.

Here, we treated differentiated mouse P19 neurons (P19N) derived from embryonal carcinoma cells with WT BoNT/A and BoNT/A-WFY at physiologically relevant concentrations ranging from 0.3 to 300 pM. After 2 h of treatment, BoNTs were washed away and the P19Ns were collected and lysed at 4, 8, 12, and 24 h post-treatment and analyzed by Western blot to quantify SNAP-25 cleaved by BoNT/A (Fig. 4b and Supplementary Fig. 4d). Intriguingly, we found that BoNT/A-WFY displayed a faster onset of action (EC_50_ ~ 10 pM) compared to the WT BoNT/A (EC_50_ ~ 300 pM) at 4 h. Over time, the WT BoNT/A gradually narrowed the gap, with EC_50_ values of ~3 pM for WFY and ~30 pM for the WT at 8 h, and ~2 pM for WFY and ~20 pM for the WT at 12 h. By 24 h, ~1 pM BoNT/A-WFY and ~5 pM WT BoNT/A each achieved ~50% SNAP-25 cleavage (Fig. 4c). In general, 3 pM of BoNT/A-WFY yields comparable SNAP-25 cleavage as 30 pM of WT BoNT/A at 4, 8, 12 h and 24 h post-treatment (Fig. 4d). Since the WFY mutations—confined to the belt—do not affect receptor binding and uptake, LC enzymatic activity, and most other features of BoNT/A, the faster SNAP-25 cleavage by BoNT/A-WFY compared to BoNT/A WT at similar concentrations indicates more rapid translocation and earlier action of LC, ultimately resulting in a quicker onset of its activity.

### The belt-buckle can be targeted to neutralize BoNT/A

**Fig. 5:**
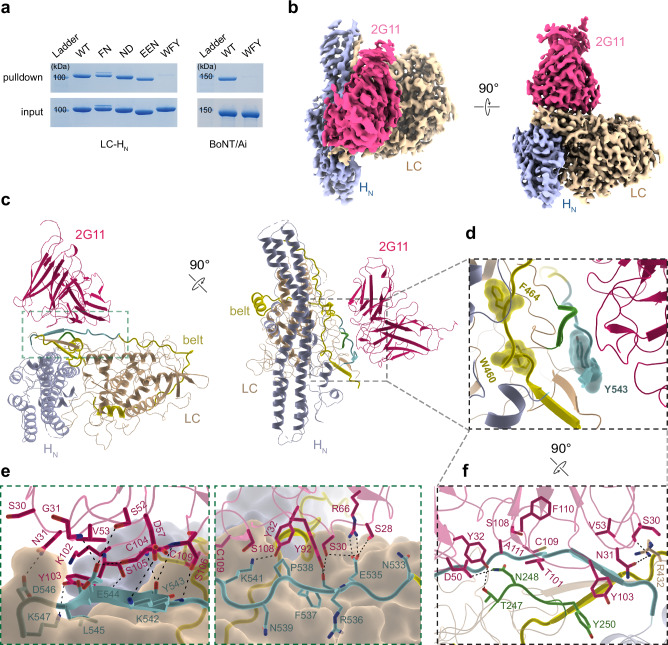
Neutralizing antibody 2G11 recognizes the belt-buckle area on BoNT/A. **a** The binding of 2G11 to BoNT/A variants were examined by a pull-down assay. Left panel: LC-H_N_/A variants; Right panel: full-length inactive BoNT/Ai and BoNT/Ai-WFY. Representative image from one of three independent experiments is shown. **b** Cryo-EM map of the BoNT/Ai–2G11 complex in two different views. LC/A, H_N_A, and the Fab fragment of 2G11 are colored wheat, blue, and hot pink, respectively. **c** Cartoon representation of the structure of the BoNT/Ai–2G11 complex. The color scheme follows that of (**b**), with the belt highlighted in yellow. The 2G11-interacting regions on the belt and LC/A are colored cyan and green, respectively. **d** A close-up view into the BoNT/Ai–2G11 interface, with the three residues mutated in the WFY variant shown as sticks/surfaces. **e**, **f** Close-up views into the BoNT/Ai–2G11 interface involving a long stretch on the belt (N533–K547) (**e**) and a loop in LC/A (T247–Y250) (**f**). The key interacting residues are shown as sticks, while the rest of BoNT/Ai is displayed as a surface.

Since destabilizing the belt-buckle speeds up BoNT/A’s action, we explored whether stabilizing it—such as by antibody binding—could serve as a countermeasure to delay or suppress its toxicity. Accordingly, we searched the literature for antibodies that may specifically recognize the belt-buckle of BoNT/A. This led us to focus on a potent human monoclonal neutralizing antibody (mAb), 2G11, one of the three mAbs included in an antitoxin drug product neutralizing BoNT/A currently in clinical trials (Supplementary Table 2). Prior studies suggest that it recognizes a 3D composite epitope involving both LC and H_N_^32–35^, suggesting it could potentially engage the belt-buckle. Leveraging our belt-loosening mutants of BoNT/A described above, we investigated whether these mutations could impact 2G11 binding using a pull-down assay. Intriguingly, we found that 2G11 did not recognize the WFY mutant, neither within the LC-H_N_ fragment nor the inactive BoNT/Ai holotoxin, while retaining WT-like binding to other LC-H_N_/A mutants (Fig. 5a). This finding suggests that the belt-buckle of BoNT/A is part of the neutralizing epitope of 2G11, which was disrupted in the WFY mutant.

### Stabilizing the belt-buckle by antibodies effectively neutralizes BoNT/A

To delve deeper into the neutralizing mechanism of 2G11 and the functional relevance of the belt-buckle, we determined the cryo-EM structure of BoNT/Ai in complex with the Fab fragment of 2G11 (Supplementary Fig. 7). Processing of the cryo-EM data yielded a high-quality electron density map for the LC-H_N_ fragment of BoNT/Ai and the variable light chain (V_L_) and heavy chain (V_H_) of 2G11 at 3.06 Å resolution (Fig. 5b, c). The densities for the H_C_ domain of BoNT/Ai and the constant light chain and heavy chain of 2G11 were not sufficient for model building due to high structural flexibility, but they are outside the well-defined 2G11–BoNT/A interface.

The cryo-EM structure clearly shows that 2G11 binds at the junction of LC/A and H_N_A around the belt-buckle area (Fig. 5c). Detailed structural analysis reveals that 2G11 recognizes two distinct regions on BoNT/Ai. First, 2G11 binds to an extended C-terminal stretch of the BoNT/A belt (N533–K547) and “locks” it into the buckle area, burying an interface of ~569 Å². This interface involves an extensive network of hydrogen bonds, salt-bridges, and hydrophobic interactions, with 13 of the 15 residues in this segment of the belt directly interacting with the V_H_ and V_L_ of 2G11, securing the belt in place (Fig. 5d, e and Supplementary Table 3). Notably, Y543 in this area of the belt directly interacts with K102, Y103, C104, and C109 on the V_L_ of 2G11. Mutating Y543, as seen in the WFY mutant, can significantly impair 2G11 binding to BoNT/A. Complementing its interactions with the belt, 2G11 also binds a small loop (T247–Y250) within LC/A through two hydrogen bonds and several hydrophobic interactions (Fig. 5f and Supplementary Table 3). In addition, residue R432 in the buckle of BoNT/A is engaged in hydrogen bonds and hydrophobic interactions with residues S30, N31, V53, and G54 on the V_H_ of 2G11 (Fig. 5f). Collectively, 2G11 binding significantly enhances the stability of the LC and belt-buckle of BoNT/A.

The overall structure of the 2G11-bound LC-H_N_/A closely resembles its free form, as observed in BoNT/A holotoxin (PDB: 3BTA) or the isolated LC-H_N_/A (PDB: 2W2D), with root mean square deviations (RMSDs) of ~0.704 Å and ~1.079 Å, respectively (Supplementary Fig. 8a). However, the loop T247–Y250 in LC/A, which is recognized by 2G11, is either too flexible to be observed (PDB: 2IMC) or exhibiting different orientations (PDB: 1E1H and 1XTF) in several structures of the isolated LC/A (Supplementary Fig. 8b–d)^49–51^. Notably, this loop in LC/A is sandwiched between the N- and C-terminal segments of the belt—where residues W460/F464 and Y543 are located, respectively—in the context of LC-H_N_/A or the holotoxin. Such interactions mutually stabilize the belt and this loop, which may explain the increased flexibility of this loop in the isolated LC lacking the belt. Conversely, when stabilized by 2G11, this loop in LC/A can relay structural support to reinforce the stability of the belt. Interestingly, we found that the substitution of A50 by D50 during affinity maturation of 2G11, derived from its parental antibody ING1^32^, significantly contributes to its enhanced affinity. This is primarily attributed to D50 on the V_L_ of 2G11 forming a hydrogen bond with N248 in this loop of LC/A, while also establishing a salt bridge with R100 on the V_H_ of 2G11 to stabilize the V_L_–V_H_ interactions.

Taken together, these structural findings demonstrate that 2G11 effectively stabilizes the structural integrity of the LC and belt-buckle in BoNT/A, preventing conformational changes in this area that are prerequisites for LC unfolding and translocation. In contrast, the engineered WFY mutations loosen the belt-buckle, not only disrupting 2G11 binding, but also facilitating pH-dependent conformational changes in this area.

Remarkably, we discovered that the binding mode of 2G11 to BoNT/A closely resembles that of a neutralizing single-domain camelid antibody (VHH), JLE-E9, in its interaction with BoNT/E, after analyzing a large number of crystal structures of BoNTs in complex with diverse VHHs in the literature^36,37,52,53^. In fact, JLE-E9 binds to the belt-buckle area on BoNT/E, with its binding site on BoNT/E almost completely overlapping that of the V_H_ of 2G11 on BoNT/A, suggesting that they share similar neutralizing mechanisms (Supplementary Fig. 9)^36^. Consistently, our prior studies demonstrate that JLE-E9 binding stabilizes the structure of LC-H_N_/E, thereby inhibiting its conformational changes induced by acidic pH^36^. These two neutralizing antibodies were developed using completely different strategies, yet both ultimately target the homologous belt-buckle in these two distinct BoNT serotypes. These findings suggest that the belt-buckle is likely one of the most vulnerable parts of BoNTs, serving a conserved functional role.

### BoNT/A-WFY can be neutralized by the available antitoxins

Besides 2G11, the other two mAbs, CR2 and RAZ1, in the antitoxin mixture currently tested in clinical trials specifically recognize H_C_A, and their epitopes are not affected by the WFY mutations located in the belt^24,33,35,54^. Therefore, they should recognize and neutralize BoNT/A-WFY as effectively as the WT toxin. Our prior studies show that a combination of two neutralizing antibodies protected mice against BoNT/A intoxication with very high lethal dosages (up to 10,000 LD_50_)^34,55^. Therefore, BoNT/A-WFY should be readily neutralizable by the antitoxin mixture.

In addition, we checked neutralization of BoNT/A-WFY vs WT by the FDA-approved heptavalent equine botulism antitoxin (BAT^®^) in a variation of the MPN assay for detecting BoNT-neutralizing antibodies (BNA), referred to as the MPN-BNA assay. Dosages of the WT BoNT/A and BoNT/A-WFY displaying similar biological activity were pre-incubated with BAT^®^, and the BoNT–BAT mixtures were tested in the MPN-BNA assay. The paralytic halftime for BoNT/A WT was prolonged from 66 min without BAT^®^ to 163 min with BAT^®^, corresponding to 98% neutralization of its biological activity. Similarly, BoNT/A-WFY showed a comparable prolongation of paralytic halftime from 70 min without BAT^®^ to 167 min with BAT^®^, resulting in 94% neutralization of its biological activity (Fig. 4e). These results demonstrate that the WFY mutations, confined to the belt, have minimal structural impact on BoNT/A and do not permit escape from neutralization by the licensed polyclonal antitoxin.

## Discussion

It is well documented that BoNT/E takes effect faster than BoNT/A and BoNT/B^4–11^. However, both BoNT/A and BoNT/E exploit SV2 for neuron binding and entry^19–25^ and cleave SNAP-25 with similar activity^9^. Moreover, BoNT/E is less potent than BoNT/A, likely due to its weaker receptor binding affinity and differing specificity for SV2 isoforms compared to BoNT/A^19,21,23^. Therefore, it was perplexing to comprehend how BoNT/E can act more quickly than BoNT/A. Here, we demonstrate that the greater flexibility of the belt of BoNT/E contributes to the rapid translocation of its LC to the cytosol compared to BoNT/A and BoNT/B by lowering the energy requirement for acidic pH-induced LC unfolding and delivery across the H_N_ channel. Furthermore, we identify a regulating checkpoint in the belt, the belt-buckle, which modulates the loosening of the belt constraint on the LC-H_N_ fragment in response to vesicle acidification. The belt-buckle can be locked by an antibody, restricting conformational changes in this area and resulting in effective toxin neutralization. In contrast, destabilizing the belt-buckle of BoNT/A through protein engineering enhances the pH sensitivity of LC-H_N_/A, leading to accelerated onset of action.

The belt is known as a surrogate pseudo-substrate inhibitor of the LC protease, which embraces the LC and stabilizes its compacted conformation^31^. In addition, a prior study suggests that the belt of BoNT/A modulates the pH-dependent conformational change and membrane insertion of H_N_A^44^. Here, we demonstrate that the belt of BoNT/E has a more flexible conformation than that of BoNT/A and BoNT/B. To accommodate the loose belt, BoNT/E evolved to adopt a distinct closed-wing conformation at neutral pH so H_C_E can provide additional support to stabilize the belt. Contrary to the prevailing views that the closed-wing conformation of BoNT/E is faster than the open form of BoNT/A and BoNT/B in LC translocation^9,30^, our cryo-EM studies reveal that BoNT/E actually opens up at acidic pH, taking an ensemble of open conformations (Fig. 2). As a result, the BoNT/E belt turns even looser when H_C_E moves away from its LC-H_N_ moiety. These findings suggest that releasing the constraint of the belt at acidic pH may prime the LC-H_N_ for subsequent unfolding and translocation. This may allow quicker translocation and earlier action of LC/E in the cytosol, eventually leading to quicker onset of symptoms. Interestingly, when BoNT/E encounters an acidic environment in the GI tract during oral intoxication, it is “locked” by NTNH/E in the M-PTC, with part of its belt protected by the nH_C_ domain of NTNH/E^41^, thereby protecting the toxin against the harsh GI environment. Future investigation is warranted to elucidate the mechanisms by which BoNTs sense environmental pH to coordinate their structure and function.

Building on these insights, we explored whether we could exploit this distinct feature of BoNT/E to speed up the onset of action of BoNT/A by loosening its belt through protein engineering. To this end, we engineered several variants of LC-H_N_/A, introducing mutations at selected regions of the belt to disrupt its interactions with LC/A, while avoiding changes to other parts of the toxin. Interestingly, we found that most of these mutations did not show any major effects on the stability or pH sensitivity of LC-H_N_/A except for the WFY mutations located in the belt-buckle. Mutating these three key LC/A-interacting residues on the belt increases the flexibility of the local structure in this area, as evidenced by the loss of antibody 2G11 binding, which specifically recognizes this area on BoNT/A. As the loosened belt reduces its constraint on the LC and H_N_, LC-H_N_/A-WFY was more sensitive to acidic pH, unfolding more rapidly and extensively than the WT protein (Fig. 3e, f). Remarkably, in contrast to the belt-loosening effect of the WFY mutant, we found that two antibodies targeting BoNT/A and BoNT/E, respectively, neutralize the toxins by stabilizing their belt-buckles (Fig. 5 and Supplementary Fig. 9), further establishing the belt-buckle as a critical regulating checkpoint.

Consistent with our structural and biochemical findings, BoNT/A holotoxin carrying the WFY mutations took effect faster than the WT toxin on neurons, as evidenced by its superior EC_50_ for SNAP-25 cleavage at each time point examined (Fig. 4b–d). These findings provide further evidence that the WFY mutations—confined to the belt-buckle—do not compromise receptor binding, LC activity, LC intracellular half-life (a key determinant of duration of action), or other intrinsic features of BoNT/A. Therefore, BoNT/A-WFY holds potential as a starting point to develop fast-acting and still long-lasting BoNT/A, meeting the same therapeutic and aesthetic needs as the conventional BoNT/A while offering the added advantage of rapidly achieving functional outcomes. A similar strategy could be applied to engineer other BoNT serotypes towards fast onset, since the regulatory mechanism of the belt-buckle is likely conserved across BoNTs. These next-generation BoNTs will offer distinct opportunities for treating neurologic conditions requiring prompt therapy and timely evaluation of treatment outcomes, as well as for aesthetic customers seeking rapid results. Conversely, our studies identify the belt-buckle as the Achilles’ heel of BoNTs with conserved functional roles, which could be explored for the development of medical countermeasures.

## Methods

### Ethics statement

The MPN assay (project license no. 2018/209) was performed in Dr. Rummel’s lab in Germany according to §4 Abs. 3 (killing of animals for scientific purposes, German animal protection law (TSchG)). The number of animals sacrificed by trained personnel before the dissection of organs is reported annually to the animal welfare officer of the Central Animal Laboratory and to the respective authority. Female mice were used for this study, but no sex-dependence observed in previous in-house studies.

### Cloning, expression, and purification of recombinant proteins

The gene encoding the LC-H_N_ of BoNT/A1 (residues 1–875) was cloned into a modified pET28a vector with a 6×His/SUMO tag introduced to the N-terminus and a twin-strep II tag at the C-terminus. A peptide linker containing a thrombin cleavage site (ASLVPRGSGGSAAA) was inserted between residues S441 and L442. The gene encoding BoNT/Ai carrying two catalytically inactivating mutations (R363A/Y366F)^56,57^ was cloned to a modified pET22b vector with an N-terminal twin-strep II tag and C-terminal 6×His tag. The gene encoding BoNT/Ei carrying three catalytically inactivating mutations (H212A/E213A/H216A) was cloned into a pGEX vector with an N-terminal GST-tag followed by a 3C cleavage site. All the mutants were obtained by QuikChange PCR and verified by sequencing.

The recombinant proteins were expressed in *E. coli* strain BL21-star (Invitrogen). The transformed cells were cultured at 37 °C in Luria Broth medium supplemented with the required antibiotics. Protein expression was induced by adding 1 mM isopropyl-β-D-thiogalactopyranoside (IPTG) when the cell density (OD_600_) reached ~0.8. The temperature was then reduced to 18 °C, and the protein expression continued at 18 °C for 18 h. Cells were harvested by centrifugation and stored at −80 °C for future use.

The cell pellets were lysed by sonication. The LC-H_N_/A variants were purified by Ni-NTA affinity resins (Thermo Fisher) in a buffer containing 50 mM Tris, pH 8.5, 400 mM NaCl, and 30 mM imidazole. The bound proteins were eluted with a similar buffer containing 400 mM imidazole. The His/SUMO tag was cleaved by SUMO-protease. These proteins were further purified using Strep-Tactin (IBA) affinity resins. The bound proteins were eluted by 20 mM HEPES, 150 mM NaCl, 50 mM D-biotin, pH 8.5, and further purified by gel filtration using a Superdex-200 increase 10/300 GL column (Cytiva) in 20 mM Tris, pH 8.5, and 250 mM NaCl. The peak fractions were pooled and concentrated to ~5 mg/ml and stored at −80 °C until use. The inactive full-length BoNT/Ai and BoNT/Ai-WFY were purified using a similar protocol. BoNT/Ei was purified as previously described^42^. The purified BoNT/Ei was dialyzed against a buffer containing 50 mM MES, pH 5, and 100 mM NaCl for structural studies.

The wild-type and mutated recombinant full-length activated BoNT/A1 were produced under biosafety level 2 containment (project number GAA A/Z 40654/3/123/3) in Dr. Rummel’s lab in Germany, using the *E. coli* BL21 DE3 strain, as previously described^45^. All mutations were generated by two-step PCR and verified by DNA sequencing. BoNT/A WT and WFY, both carrying a thrombin recognition site in the linker connecting LC and H_N_ and a thrombin cleavable C-terminal Strep/6×His-tag, were purified using a Co^2+^-Talon matrix (Takara Bio Europe S.A.S., France) and eluted with PBS, pH 7.4, and 250 mM imidazole. For proteolytic activation and removal of the affinity tag, BoNTs were incubated for 16 h at room temperature with 0.01 U bovine thrombin (MP Biomedicals Germany GmbH, Germany) per μg of BoNT. Subsequent gel filtration (Superdex-200 SEC; Cytiva, Germany) and negative IMAC using a Co^2+^-Talon column was performed in phosphate-buffered saline (pH 7.4). For storage, BoNTs were shock-frozen in liquid nitrogen and stored at −80 °C. Protein concentration was determined by A280 spectrometry. Homogeneity of proteins was verified by subjecting 320 µg of BoNT/A WT and WFY to analytical SEC (Superdex 200 Increase 10/300 GL). Purity and degree of activation were checked by 10% SDS-PAGE analysis.

### Negative-stain EM sample preparation, data acquisition, and processing

For negative-stain EM sample preparation, 3.5 µl of BoNT/Ai-WT and BoNT/Ai-WFY at a concentration of 25 µg/ml was applied to glow-discharged carbon-coated copper grids (CF200-CU, 200 mesh). After incubation for 1 min, excess sample was blotted away, and the grids were stained with 2% (w/v) uranyl acetate for 1 min. The grids were then air-dried before imaging.

Negative-stain EM data were collected on a JEOL JEM-2100F transmission electron microscope operated at 200 kV. Images were recorded at a nominal magnification of 50 kX, corresponding to a pixel size of 1.7 Å, using a Gatan OneView detector. Approximately 200 micrographs were collected for each sample with a defocus range of −1.7 to −3.1 μm.

All acquired micrographs were processed using cryoSPARC v4.4.1^58^. Contrast transfer function (CTF) parameters were estimated using patch-based CTF estimation. Particles were automatically picked using the Blob Picker and then extracted for subsequent 2D classification. Multiple rounds of 2D classification were performed to remove junk particles and generate representative class averages.

### Cryo-EM sample preparation and data acquisition

For cryo-EM sample preparation, 4 μl of BoNT/Ei was applied at concentrations of ~0.5 mg/ml to glow-discharged holey carbon grids (Quantifoil Grid R1.2/1.3 Cu 300 mesh). The grids were blotted for 1 s using an FEI Vitrobot plunger at 10 °C and 100% humidity, and then plunge-frozen in liquid ethane cooled by liquid nitrogen. The frozen grids were stored in liquid nitrogen before use.

High-resolution Cryo-EM data were collected on a Titan Krios electron microscope operating at 300 kV, equipped with a Gatan K3 direct electron detector at the Pacific Northwest Center for Cryo-EM (PNCC). All the datasets were collected using similar parameters. The microscope was operated at a magnification of 29 kX, resulting in a pixel size of 0.788 Å. All images were automatically recorded using SerialEM^59^. Movies were obtained at an accumulated dose of 50 e^–^/Å^2^ with defocus ranging from −0.7 to −2.3 μm, with 65 frames per movie stack.

The antibody 2G11 was provided by Dr. James Marks^32^. The Fab fragment of 2G11 was generated using immobilized papain (Thermo Fisher Scientific), following the manufacturer’s protocol. To prepare the BoNT/Ai–Fab complex, purified BoNT/Ai was mixed with the Fab fragment at a molar ratio of 1:1.5 and incubated on ice for 2 h. The complex was subsequently purified using a Superdex 200 10/300 GL column in a buffer containing 20 mM HEPES, pH 7.5, and 150 mM NaCl. The purified complex was concentrated to ~3.5 mg/ml for cryo-EM analysis.

For cryo-EM data collection, 4 µl of purified BoNT/Ai–Fab complex was applied at concentrations of ~0.2 mg/ml to glow-discharged holey carbon grids (Quantifoil Grid R1.2/1.3 Cu 300 mesh). The grids were blotted for 2 s using a Thermo Fisher Vitrobot Mark IV at 10 °C and 100% humidity, and then plunge-frozen in liquid propane cooled by liquid nitrogen. The frozen grids were stored in liquid nitrogen before use. High-resolution cryo-EM data were collected on a Titan Krios electron microscope operating at 300 kV and equipped with a BioContinnum energy filter with Gatan K3 direct electron detector at PNCC. All the datasets were collected using similar parameters. The microscope was operated at a magnification of 105 kX, resulting in a pixel size of 0.8266 Å. All images were automatically recorded using SerialEM^59^. Movies were obtained at an accumulated dose of 50 e^–^/Å^2^ with defocus ranging from −0.8 to −2.2 μm, with 59 frames per movie stack.

For cryo-EM data collection, 4 µl of purified BoNT/Ai-WFY supplemented with 0.05% OG (n-Octyl-β-D-Glucoside, Anatrace) was applied at concentrations of ~2.5 mg/ml to glow-discharged holey carbon grids (Quantifoil Grid R1.2/1.3 Cu 300 mesh). The grids were blotted for 3 s using a Thermo Fisher Vitrobot Mark IV at 10°C and 100% humidity, and then plunge-frozen in liquid ethane cooled by liquid nitrogen. The frozen grids were stored in liquid nitrogen before use. Cryo-EM data were collected on a Titan Krios electron microscope operating at 300 kV and equipped with a Selectris X energy filter with Falcon 4i camera at the Electron Imaging Center for Nanosystems (EICN) at the University of California, Los Angeles’ California for NanoSystems Institute (CNSI). The microscope was operated at a magnification of 130 kX, resulting in a pixel size of 0.97 Å. All images were automatically recorded using EPU. Movies were obtained at an accumulated dose of 50 e^–^/Å^2^ with defocus ranging from −0.8 to −2.8 μm.

### Data processing and structure determination

All acquired movies were processed using cryoSPARC v4.4.1^58^. The movies were motion-corrected and dose-weighted using the patch-based motion correction module. The contrast transfer function (CTF) of the resulting images was then subject to patch-based estimation. Particles were auto-picked using blob picker and template picker.

For the BoNT/Ei dataset, the particles were processed with rounds of 2D classification, and the selected particles were used for ab initio reconstruction, followed by further heterogeneous refinement.

For the LC-H_N_ class, the M-PTC form, and other open classes of BoNT/Ei, the selected particles were used for non-uniform refinement. For the open classes, the selected particles were further processed with 3D classification, 2D classification, followed by further non-uniform refinement.

For the BoNT/Ai–Fab complex dataset, after rounds of 2D classification, the selected particles were used for ab initio reconstruction, followed by further non-uniform refinement.

For the BoNT/Ai-WFY dataset, after rounds of 2D classification, the selected particles were used for ab initio reconstruction, followed by heterogeneous refinement and further homogeneous refinement.

For all processed EM maps, the average resolution was given by the Fourier Shell Correlation criterion (FSC 0.143). CryoSPARC was used to determine local resolution and sharpen the maps. The data processing statistics of all datasets were summarized in Supplementary Figs. 2, 6, 7, and Supplementary Table 1.

For model building of the LC-H_N_/E structure and the holotoxin in the M-PTC form, the reported structures of LC-H_N_/E (PDB: 7K84)^36^ and the M-PTC/E (PDB: 9ARJ)^42^ were used to fit into the cryo-EM maps in UCSF ChimeraX^60^, respectively.

For model building of the BoNT/Ai–Fab complex, the reported structure of BoNT/A (PDB: 3BTA)^27^ and Alphafold3^61^ predicted Fab structure were used to fit into the cryo-EM maps in ChimeraX.

The initial structural models were subject to iterative refinement using Coot (v0.8.1)^62^ and real-space refinement using Phenix (v1.20.1)^63^. The statistics of data collection and structural refinement for all the structures reported in this manuscript are summarized in Supplementary Table 1. Figures were generated using PyMOL (http://www.pymol.org) and ChimeraX. An analysis of the interaction surface was carried out using PISA.

### ANS (8-anilinonaphthalene-1-sulfonic acid) binding assay

LC-H_N_/A variants (1 µM) were incubated with 200 µM of ANS for 15 min. Buffers were formulated with 50 mM StockOption pH buffer kit with pH ranging from 4.4 to 8.4 (Hampton Research, HR2-241), supplemented with 250 mM NaCl. Fluorescence intensities were recorded at 25 °C using a Molecular Devices SpectraMax M2e spectrophotometer with excitation at 366 nm and emission at 482 nm. The fluorescence intensity was normalized to that of wild-type LC-H_N_/A at pH 8.4. Error bars indicate SD of three replicate measurements.

### Protein-melting assay

The thermal stability of LC-H_N_/A variants was measured using a fluorescence-based thermal shift assay on a StepOne real-time PCR machine (Life Technologies). Buffers were formulated with 50 mM StockOption pH buffer kit with pH ranging from 4.4 to 8.4 (Hampton research, HR2-241), supplemented with 250 mM NaCl. Immediately before the experiment, proteins (~0.25 mg/ml) were mixed with the fluorescent dye SYPRO Orange (Sigma-Aldrich), and the mixtures were heated from 25 to 95 °C in a linear ramp rate of 1.5 °C/min. The midpoint of the protein-melting curve (T_m_) was determined using the analysis software provided by the instrument manufacturer. Data obtained from three independent experiments were averaged to generate the graph.

### Pull-down assay

The interactions between antibody 2G11 and BoNT/Ai or LC-H_N_/A variants were investigated using a pull-down assay. Protein A resins (Genscript) were utilized in a PBS buffer (pH 7.4) containing 0.5% Tween-20 (PBS-TW20). Antibody 2G11 was employed as bait, while BoNT/Ai or LC-H_N_/A variants served as prey. Antibody 2G11 was first incubated with protein A resins at room temperature for 1 h to allow binding. Following incubation, unbound 2G11 was removed by washing the resins twice with the PBS-TW20 buffer. The resins were then aliquoted and mixed with LC-H_N_/A or BoNT/Ai variants at approximately threefold molar excess over 2G11. The reaction mixtures were incubated at room temperature for 2 h, followed by two washes in the PBS-TW20 buffer. The bound proteins were eluted by boiling the resins in SDS-PAGE loading buffer at 95 °C for 5 min, followed by SDS-PAGE analysis and subsequent staining with BlinkBlue (InnoCyto).

### MPN assay

The MPN assay was performed employing 20–30 g Swiss mice (Janvier SA, France) as described previously^46^. Mice were euthanized by CO_2_ anesthesia and subsequently exsanguinated. The phrenic nerve hemidiaphragm tissue was explanted, placed into an organ bath, and continuously stimulated at 5–25 mA with a frequency of 1 Hz and a 0.1 ms pulse duration. Isometric contractions were transformed using a force transducer and recorded with VitroDat Online software (FMI GmbH, Germany). The time required to decrease the amplitude to 50% of the starting value (paralytic halftime) was determined. To allow comparison of the altered neurotoxicity of mutants with BoNT/A1 WT (displaying a specific biological activity of 6.87 × 10^7^ ± 1.15 × 10^7^ mouse LD_50_/mg, mean ± SD), its MPN assay dose–response-curve logarithmic function (y(BoNT/A wild-type; 0.95, 1.9, 3.8 pM) = −21.64 ln(x) + 90.194, *R*^2^ = 0.998) consisting of three concentrations determined in a minimum of five replicates was established. Accordingly, a dose–response-curve logarithmic function for the mutant (y(BoNT/A WFY; 2.5, 5.0, 10 pM) = −35.11 ln(x) + 135.17, *R*^2^ = 0.965) consisting of three concentrations determined in 3–5 replicates each was determined. Employing the inverse logarithmic functions of the dose–response curves, the theoretical concentrations of BoNT/A WT and WFY mutant causing a paralytic halftime of 75 min were calculated (2.05 pM vs 5.56 pM) and the BoNT/A WFY biological activity was expressed as relative biological activity of BoNT/A WT.

### Neutralization of BoNT by MPN-BNA assay

To test the neutralization of BoNTs by the heptavalent equine botulism antitoxin licensed in the US (BAT^®^), dosages of BoNT/A WT (3.3 pM) and BoNT/A WFY (7.5 pM) causing similar paralytic halftime, were pre-incubated with 3.5 mU/mL of BAT^®^ or without antitoxin for 60 min at 37 °C and subsequently checked for resulting paralytic halftimes in a variation of the above described MPN assay to detect BoNT-neutralizing antibodies (BNA). Resulting paralytic halftimes can be calculated into theoretical BoNT concentrations using the respective inverse functions of the dose–response curves and expressed as relative neutralization due to BAT treatment.

### P19N SNAP-25 cleavage assay

P19 embryonal carcinoma cells were differentiated to neurons by retinoic acid (RA) treatment as previously described with minor changes^64^. Briefly, P19 cells were maintained in an undifferentiated state with a confluence of max. 90% at 37 °C with 5% CO_2_ in MEM (Thermo Fisher) supplemented with 10% FCS and a combination of penicillin (50 U/mL) and streptomycin (50 µg/mL) (MEM++). For differentiation, 1.0  × 10^6^ cells in 15 ml MEM++ were seeded on petri dishes (Sarstedt) in the presence of 5 × 10^−7^ M RA (Sigma-Aldrich). After four days of incubation at 37 °C with 5% CO_2_, growing cell aggregates, so-called embryoid bodies, were washed with 10 mL PBS, trypsinized, and plated onto poly-D-lysine-coated 24-well plates (3 µg/cm^2^ PDL, Thermo Fisher) at a density of 2.0 × 10^5^ cells/well. The medium was exchanged to Neurobasal medium (Thermo Fisher) supplemented with B-27 (Thermo Fisher) and penicillin/streptomycin (described above) (Neurobasal medium++) after 4 h when the cells show adherence. Every two days, medium was replaced with fresh Neurobasal medium++. For neuron-specific selection of differentiated P19 neurons (P19N), (8 × 10^−6 ^M) cytosine arabinoside (AraC, Sigma-Aldrich) was added to the medium four days after seeding, and experiments were conducted within eight to twelve days after seeding.

For BoNT cell-based assays, the P19N were incubated with a concentration series of BoNT/A WT and BoNT/A-WFY ranging from 0.3 to 300 pM in 500 µL Neurobasal medium++ at 37 °C and 5% CO_2_ for 2 h. Cells w/o BoNT served as negative controls. After two hours, non-bound/-endocytosed BoNT was removed, and P19N were washed with 500 µL PBS and subsequently incubated in 500 µL Neurobasal medium++ for an additional 2, 6, 10, and 22 h. Incubation was terminated by a wash step with 500 µL PBS, and subsequently P19N were incubated in 50 µL lysis buffer (200 mM NaCl, 1% NP40, 1 mM DTT, 1 mM EDTA, pH 8) for 30 min at 4 °C. P19N lysates were separated from cellular debris by centrifugation at 20,000×*g* for 10 min at 4 °C and subjected to western blot analysis. Intact and cleaved SNAP-25 (AA1-197) were detected by the mouse monoclonal anti-SNAP-25 antibody clone 71.2 (#111111; Synaptic Systems GmbH, Göttingen, Germany), recognizing an epitope present in both entities, while VAMP-2, serving as a control protein, was detected by the mouse monoclonal anti-VAMP-2 antibody clone 69.1 (#104211; Synaptic Systems GmbH, Göttingen, Germany). ECL signals were detected by the INTAS ECL ChemoStar system using the overexposure feature to maintain signal intensities in the linear range. SNAP25 signal intensities were quantified by the ImageJ software package and background subtracted. SNAP25 cleavage [%] was calculated as: [SNAP25 AA1-197] / ([SNAP25 AA1-197] + [SNAP25 AA1-206]) x 100. Results of three independent experiments were analyzed by GraphPad Prism 10 software.

### Reporting summary

Further information on research design is available in the Nature Portfolio Reporting Summary linked to this article.

## Supplementary information

Supplementary Information

Reporting Summary

Transparent Peer Review file

## Source data

Source Data

## Data Availability

The cryo-EM maps of the BoNT/Ai–2G11 complex, BoNT/Ai-WFY, LC-H_N_/E, and the classes 1–5 of BoNT/Ei at pH 5 have been deposited in the Electron Microscopy Data Bank (EMDB) under the accession codes EMD-49339, EMD-76739, EMD-46410, EMD-46409, EMD-46800, EMD-46801, EMD-46802, and EMD-46803, respectively. The atomic coordinates of the BoNT/Ai–2G11 complex, LC-H_N_/E, and BoNT/Ei at pH 5 (class 1) have been deposited in the Protein Data Bank (PDB) under accession codes 9NEY, 9CZC, and 9CZB, respectively. Other PDBs used in this paper include: 3FFZ, 3BTA, 7K84, and 9ARJ. All data supporting the findings of this study are available within the paper and the Supplementary Information. Source data are provided with this paper.
