# Supplementary material for: A belt-buckle checkpoint regulates the onset of botulinum neurotoxin intoxication
Source: Nat Commun. 2026 Jun 23;17:5562. doi: 10.1038/s41467-026-74499-7 (PMC13291336; doi:10.1038/s41467-026-74499-7)
Supplement: Supplementary file 1 — Supplementary Information [file 41467_2026_74499_MOESM1_ESM.pdf]

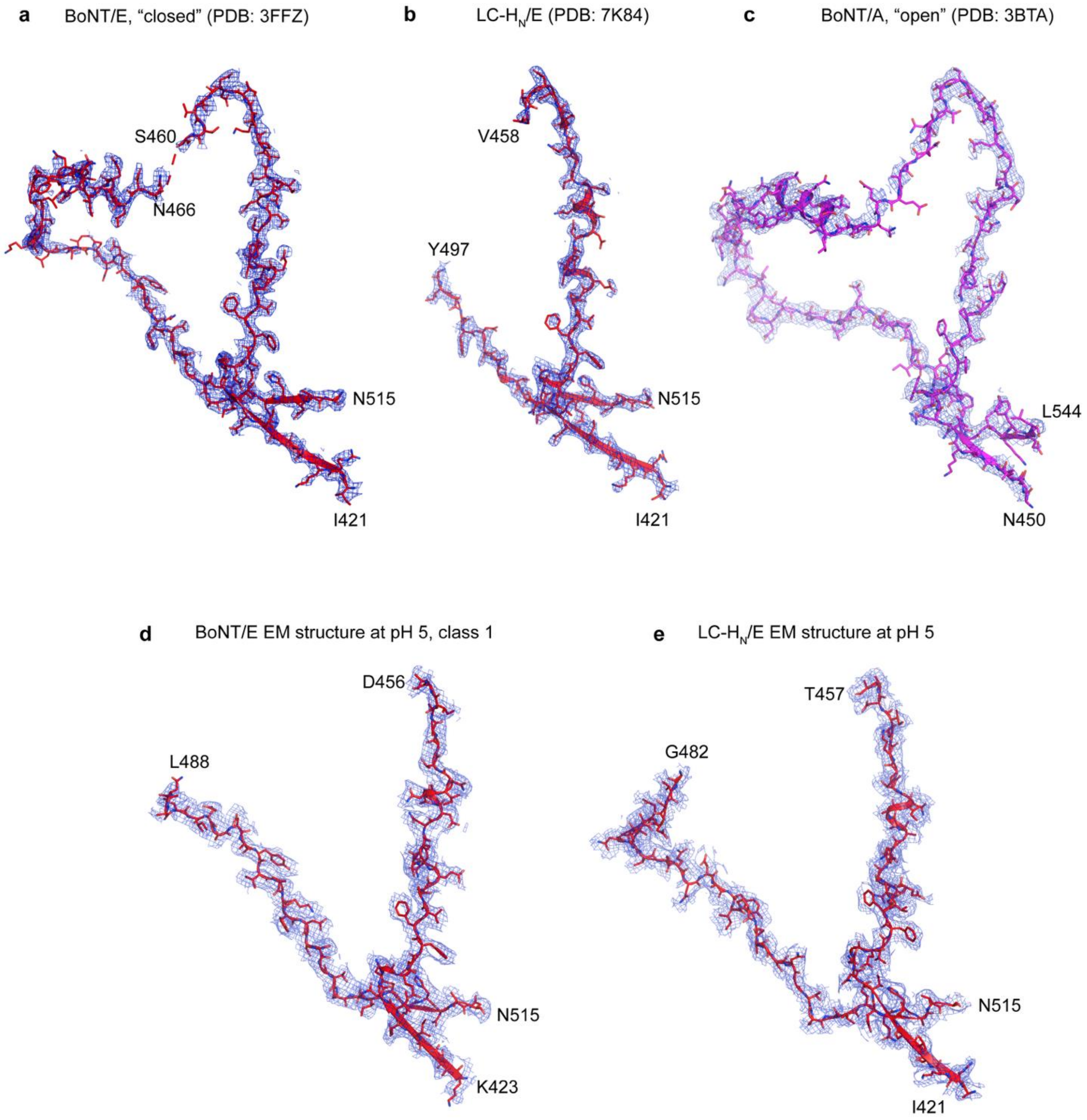

Supplementary Figure 1. Structural comparison of the belts between BoNT/E and BoNT/A under various conditions. a-c. 2Fo-Fc electron density maps (contour level = 1.2, blue mesh) of the belts in the crystal structure of BoNT/E holotoxin in the “closed” conformation (PDB: 3FFZ) (**a**), the crystal structure of LC-H$_N$/E (PDB: 7K84) (**b**), and the crystal structure of BoNT/A holotoxin in the “open” conformation (PDB: 3BTA) (**c**). **d-e.** The densities of the belt (contour level = 4.0, blue mesh) in the cryo-EM structures of LC-H$_N$/E and BoNT/Ei holotoxin at pH 5.

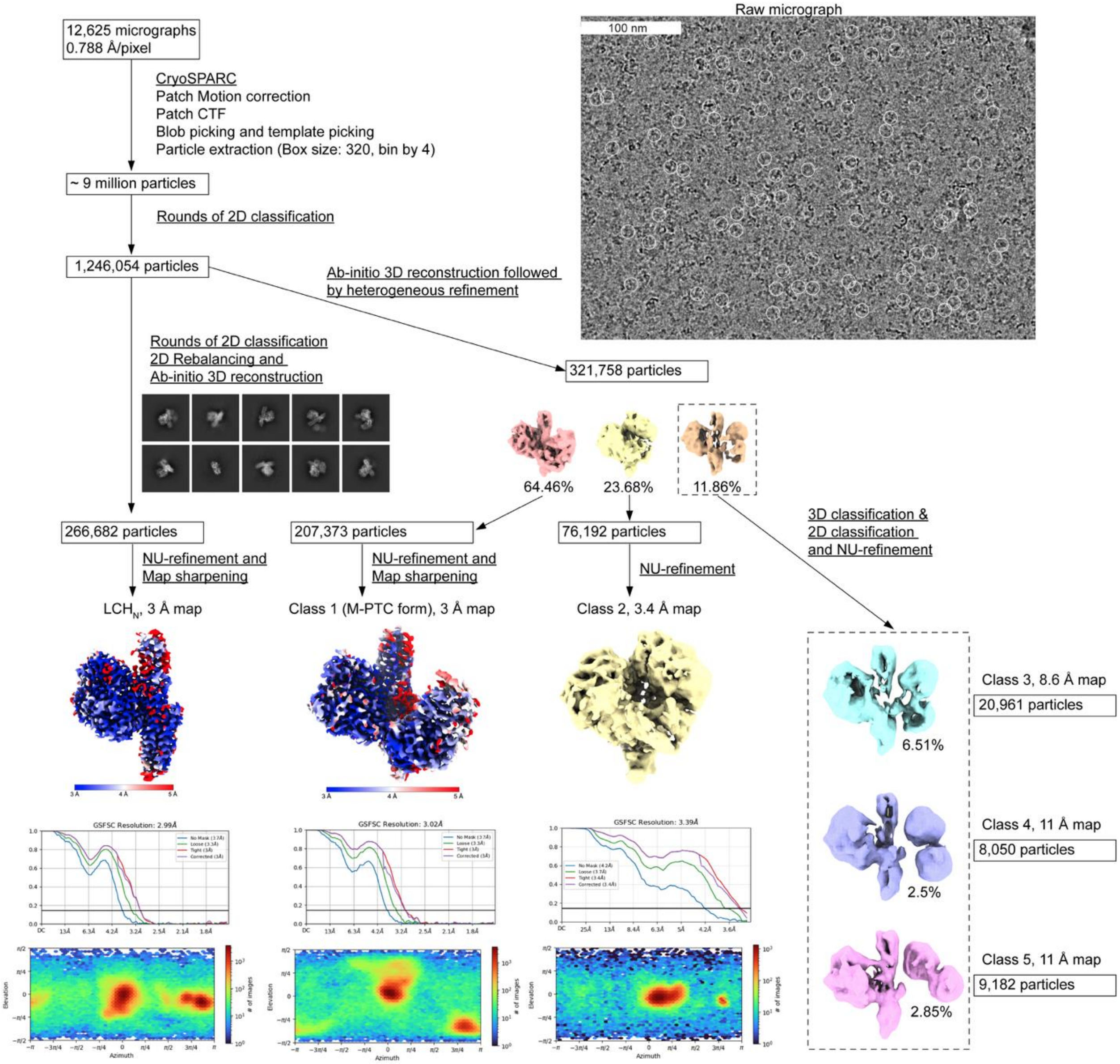

Supplementary Figure 2. Cryo-EM data processing workflow of BoNT/Ei at pH 5. Examples of a cryo-EM micrograph and 2D classes were shown in the left panels.

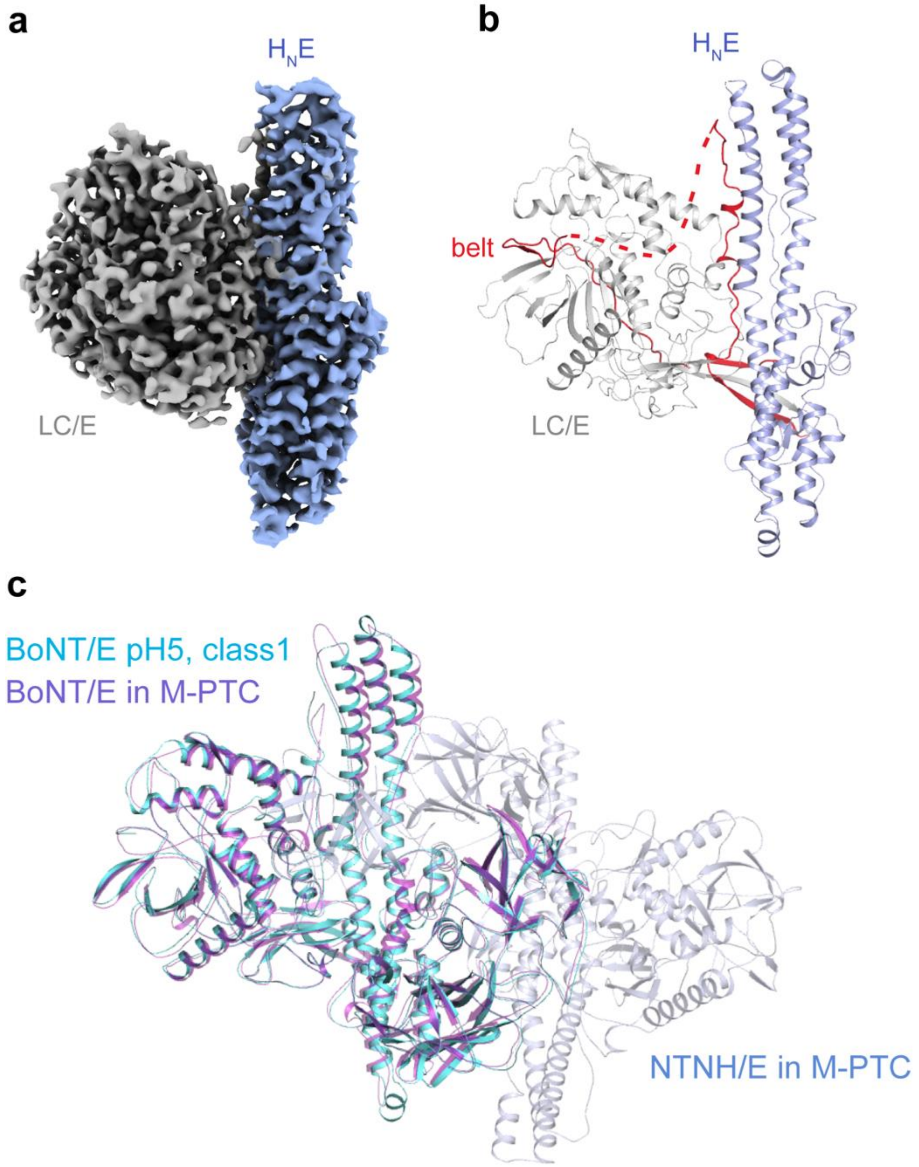

Supplementary Figure 3. Cryo-EM structures of BoNT/Ei at pH 5. a. Cryo-EM map of LC-H_N/E at 3 Å resolution. **b.** A cartoon representation of the atomic model of LC-H_N/E at pH 5. **c.** Structural superimposition of BoNT/Ei in class 1 conformation at pH 5 (cyan) with BoNT/E in the M-PTC form (purple) (PDB: 9ARJ). NTNH/E in the M-PTC is colored in gray.

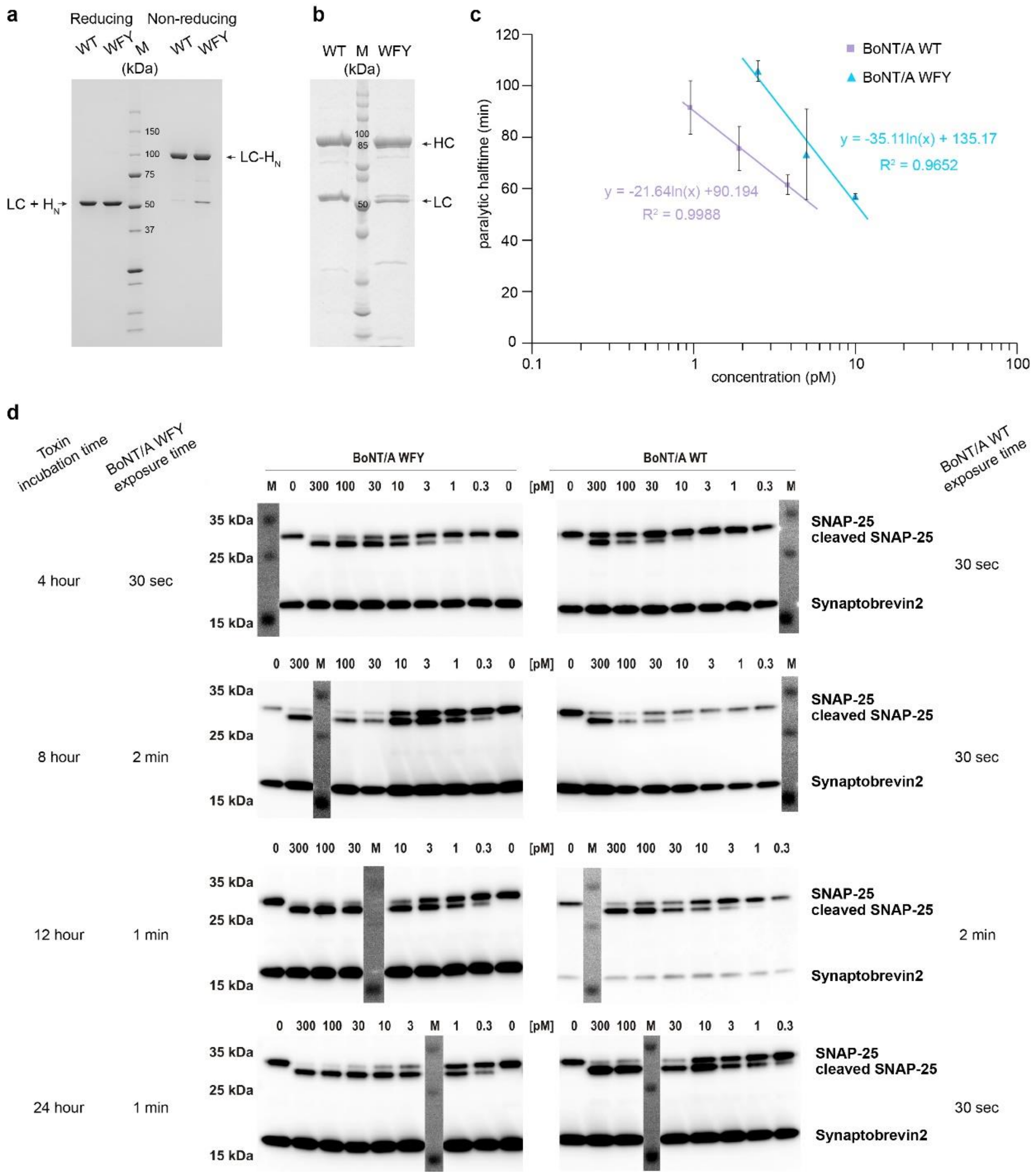

Supplementary Figure 4. Functional characterization of the engineered BoNT/A-WFY. a. Thrombin cleaved WT LC-H_N/A and the WFY mutant were examined by SDS-PAGE under reducing and non-reducing conditions. Thrombin cleavage was carried out at a 1:200 (thrombin: protein, w/w) ratio at room temperature for 2 hours. **b**, The activated BoNT/A WT and WFY resolved on a reducing SDS-PAGE, each containing a short peptide including a thrombin cleavage site inserted between K^{438} and A^{449} in the LC-HC linker. The appearance of two LC bands observed for BoNT/A-WFY may result from additional cleavage at the natural cleavage site K^{438} or another Arg/Lys upstream of the thrombin site in the linker, likely due to the increased flexibility in the belt. **c**, The biological activity of BoNT/A WT and WFY were examined using the mouse phrenic nerve hemidiaphragm (MPN) assay. **d**, Mouse P19 neurons (P19N) were treated with WT BoNT/A or BoNT/A-WFY at indicated concentrations, washed after 2 h, and subsequently subjected to Western blot analyses using a monoclonal anti-SNAP-25 antibody at 4, 8, 12 and 24 h post BoNT addition. "M" indicates the MW marker. Synaptobrevin2 was used as a control. Representative immunoblots from three independent experiments are shown.

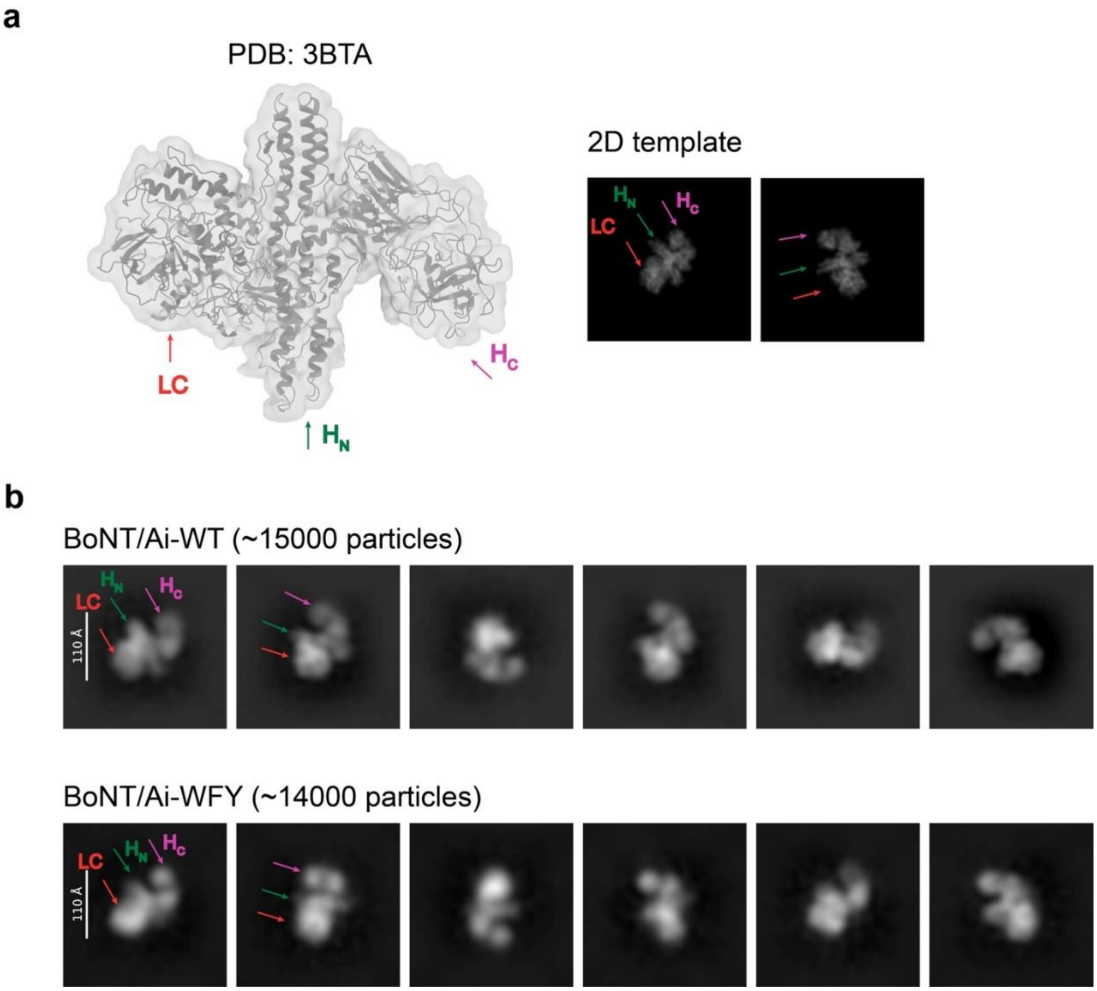

Supplementary Figure 5. Negative-stain EM analysis of BoNT/Ai-WT and BoNT/Ai-WFY. a. Structure of wild-type BoNT/A (PDB: 3BTA) highlighting the three major domains: light chain (LC, red), translocation domain (H_N, green), and receptor-binding domain (H_C, magenta). Representative 2D template views generated by CryoSPARC are shown on the right, with arrows indicating the approximate positions of each domain. **b**. Representative 2D class averages of BoNT/Ai-WT and BoNT/Ai-WFY obtained from negative-stain EM. Selected class averages are shown, with arrows marking the approximate positions of LC, H_N, and H_C.

s

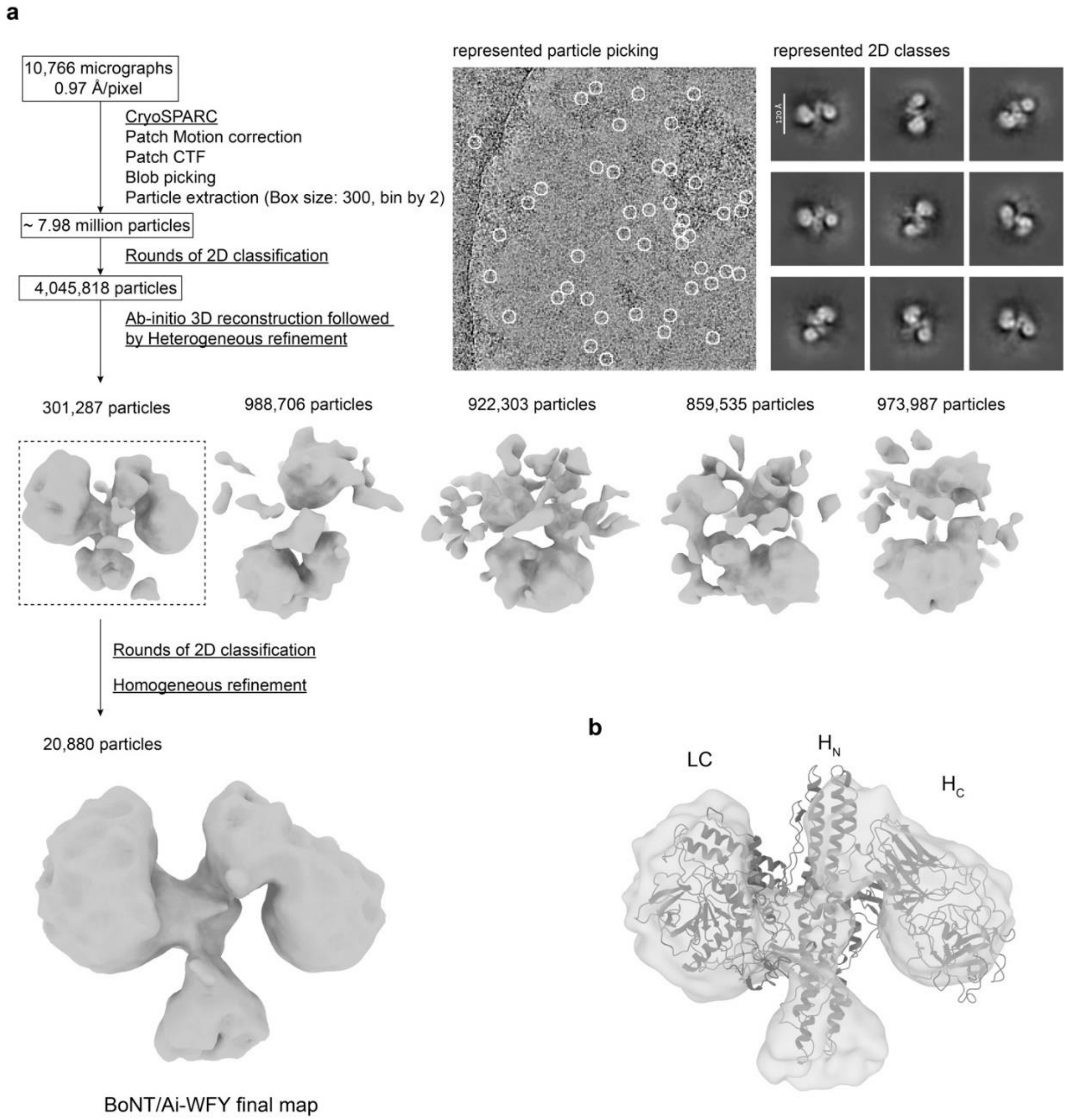

Supplementary Figure 6. The workflow of cryo-EM analysis of BoNT/Ai-WFY. a. The workflow of cryo-EM data processing of BoNT/Ai-WFY. Examples of a cryo-EM micrograph and 2D classes were shown in the right panels. **b**. The crystal structure of wild-type BoNT/A (PDB: 3BTA) is fitted into the BoNT/Ai-WFY density map to illustrate the overall domain arrangement.

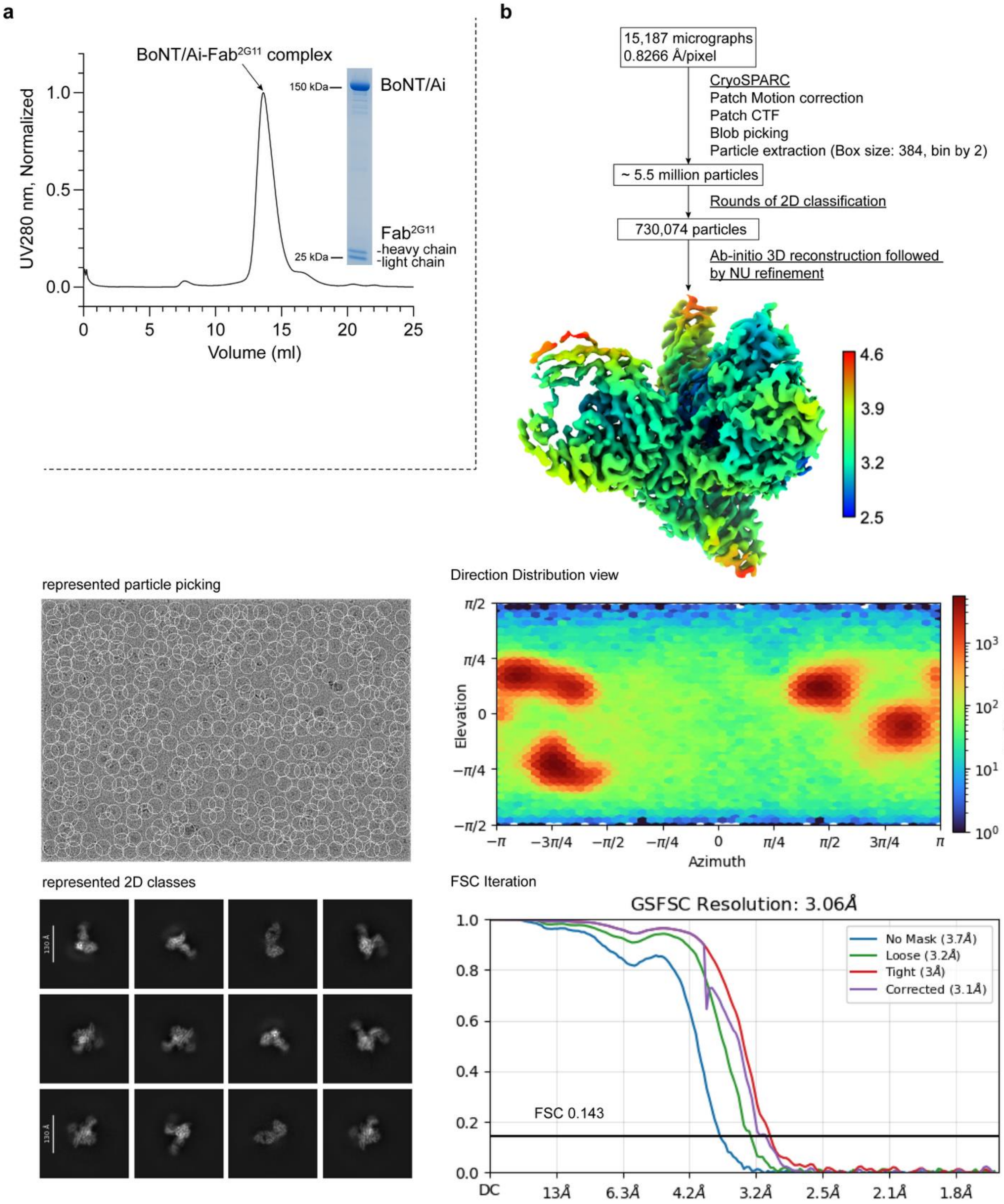

Supplementary Figure 7. The workflow of cryo-EM reconstruction of the BoNT/Ai–2G11 complex. **a.** The quality of the BoNT/Ai–2G11 complex used for cryo-EM studies was characterized by SEC and SDS-PAGE. **b.** The workflow of cryo-EM data processing and structure determination of the BoNT/Ai–2G11 complex. Examples of a cryo-EM micrograph and 2D classes were shown in the right panels.

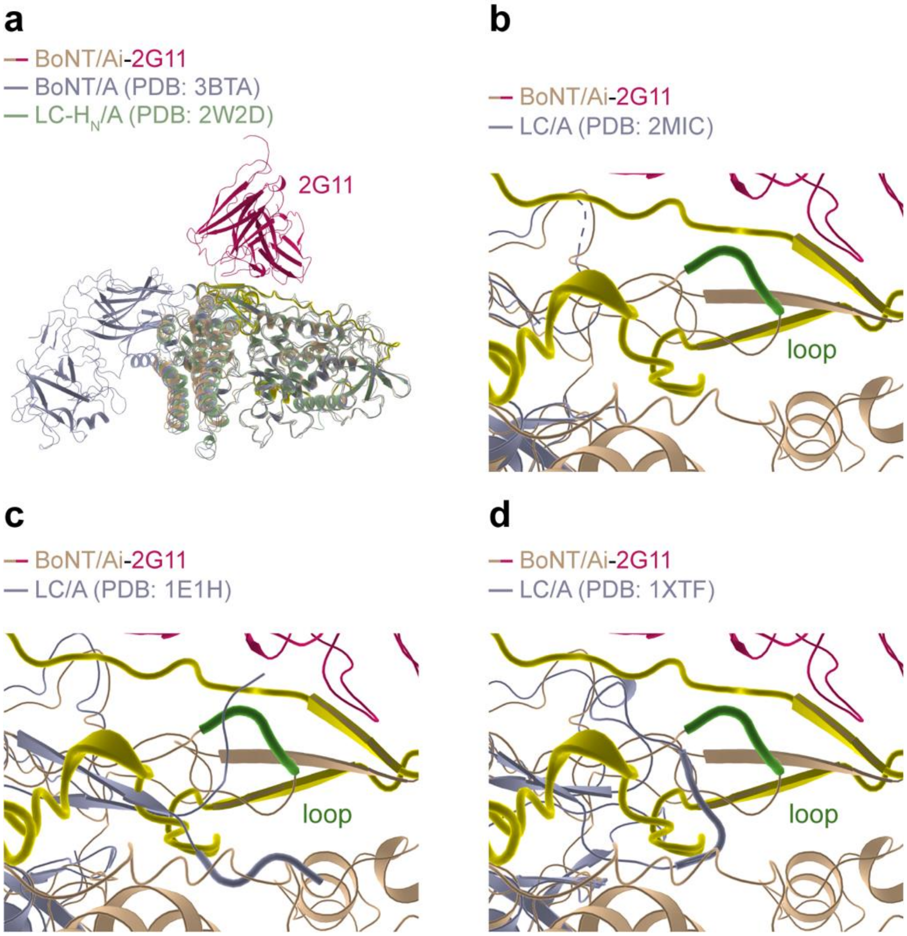

Supplementary Figure 8. Structural comparison among the 2G11-bound BoNT/Ai, the free BoNT/A, and the isolated LC/A. a. Structural superposition of the 2G11-bound BoNT/Ai (BoNT/Ai: wheat; 2G11: hot pink; belt: yellow), apo BoNT/A (light blue, PDB: 3BTA), and LC-H_N/A (light green, PDB: 2W2D). **b-d.** Structural superposition of the 2G11-bound BoNT/Ai (wheat) with three representative structures of the isolated LC/A (colored in light blue): PDBs 2IMC (**b**), 1E1H (**c**), and 1XTF (**d**). The belt in the BoNT/Ai–2G11 complex is colored in yellow, while the 2G11-binding loop in the LC is colored green. This loop is either missing (**b**); partially missing while the visible part exhibits a large conformational change (**c**), or exhibiting a large conformational change (**d**) in the isolated LC/A.

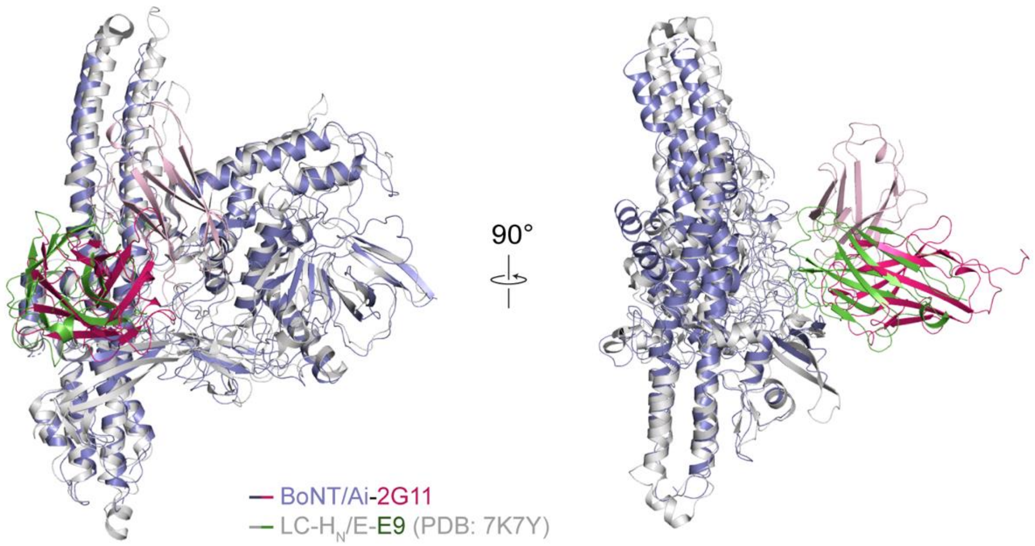

Supplementary Figure 9. Antibodies 2G11 and JLE-E9 specifically recognize the belt-buckle in BoNT/A and BoNT/E, respectively, in a similar manner. The structures of the BoNT/Ai–2G11 complex (BoNT/Ai: light blue; V_H of 2G11: hot pink; V_L of 2G11: light pink) and the LC-H_N/E–E9 complex (LC-H_N/E: grey; E9: green) were superimposed based on their LC-H_N fragments.

Supplementary Table 1. Cryo-EM data collection, refinement, and validation statistics

Structures	BoNT/Ai-2G11	BoNT/Ai-WFY	LC-H_N/E	BoNT/Ei at pH 5, class 1	BoNT/Ei at pH 5, class 2	BoNT/Ei at pH 5, class 3	BoNT/Ei at pH 5, class 4	BoNT/Ei at pH 5, class 5
EMDB code	49339	76739	46410	46409	46800	46801	46802	46803
PDB code	9NEY		9CZC	9CZB				
**Data collection and processing**								
Magnification	105,000	130,000	29,000	29,000	29,000	29,000	29,000	29,000
Voltage (kV)	300	300	300	300	300	300	300	300
Electron exposure ($e^-/Å^2$)	50	50	50	50	50	50	50	50
Defocus range (μm)	-0.8 to -2.2	-0.8 to -2.8	-0.7 to -2.3	-0.7 to -2.3	-0.7 to -2.3	-0.7 to -2.3	-0.7 to -2.3	-0.7 to -2.3
Pixel size (Å)	0.8266	0.97	0.788	0.788	0.788	0.788	0.788	0.788
Symmetry imposed	C1	C1	C1	C1	C1	C1	C1	C1
Initial particle images (No.)	5,547,260	7,981,286	1,246,054	1,246,054	1,246,054	1,246,054	1,246,054	1,246,054
Final particle images (No.)	730,074	20,880	266,682	207,373	76,192	20,961	8,050	9,821
Map resolution (Å)	3.06	7.51	3.0	3.0	3.4	8.6	11	11
FSC threshold	0.143	0.143	0.143	0.143	0.143	0.143	0.143	0.143
**Refinement**								
Initial model used (PDB code)	3BTA, AlphaFold3		7K84	9ARJ				
Model resolution (Å)	3.0		3.0	3.0				
FSC threshold	0.143		0.143	0.143				
Map sharpening B factor ($Å^2$)	-150.3		-111	-105.6				
Model composition								
Non-hydrogen atoms	8015		6214	9251				
Protein residues	1042		796	1177				
Ligands	0		0	0				
B factors ($Å^2$)								
Protein	55.84		77.12	72.4				
Ligand	0		0	0				
R.M.S. deviations								
Bond lengths (Å)	0.004		0.004	0.003				
Bond angles (°)	0.526		0.528	0.545				
Validation								
MolProbity score	2.26		2.30	2.57				
Clashscore	6.46		10.84	13.93				
Poor rotamers (%)	4.89		4.90	5.67				
Ramachandran plot								
Favored (%)	94.4		96.58	95.09				
Allowed (%)	5.6		3.42	4.91				
Disallowed (%)	0		0	0				

Supplementary Table 2. The amino acid sequence of the Fab fragment of 2G11

Heavy Chain	QVQLQQSGGGLVQPGGSLRLSCAASGFTFSNYAMTWVRQAPGKGLEWV SSISVGGSDTYYADSVKGRFTVSRDNSKNTLLLQMNSLRAEDTAVYYCAKV RTKYCSSLSCFAGFDSWGQGTRVTVSSASTKGPSVFPLAPSSKSTSGGTA ALGCLVKDYFPEPVTVSWNSGALTSGVHTFPAVLQSSGLYSLSSVVTVPSS SLGTQTYICNVNHKPSNTKVDKK
Light Chain	DVVMTQSPSSLSASVGDRVTITCRASQSISSYLHWYQQKPGKAPTLLISDA SSSQSGVPSRFSGSRFGTDFTLTISSLQPEDFATYYCQQSYSTRALTFGGG TKVEIKRTVAAPSVFIFPPSDEQLKSGTASVVCLLNNFYPREAKVQWKVDN ALQSGNSQESVTEQDSKDSTYSLSSTLTLSKADYEKHKVYACEVTHQGLS SPVTKSFNRGEC

Supplementary Table 3. Protein-protein interactions between the antibody 2G11 and BoNT/Ai

2G11	BoNT/Ai	Interaction type
V_L-Y32	T247	HB (sc-sc)
V_L-D50	N248	π-cation, vdW
V_H-T101		HB (sc-sc)
V_H-S108		vdW
V_H-C109		vdW
V_H-F110		vdW
V_H-A111		vdW
V_H-Y103		vdW
V_H-S30	Y250	vdW
V_H-N31	R432	HB (mc-sc)
V_H-V53		HB (sc-sc)
V_H-G54		vdW
V_L-S28		vdW
V_L-S28	N533	vdW
V_L-S30	E535	HB (sc-sc)
V_L-R66		HB (sc-sc)
V_L-S30		HB (sc-sc), SB
V_L-Y92	R536	HB (sc-mc)
V_L-Y92		HB (sc-mc)
V_L-Y32	F537	vdW
V_L-Y92	P538	vdW
V_H-S108		π-π, vdW
V_H-S108		vdW
V_L-Y32	N539	vdW
V_L-Y92	K541	HB (sc-sc)
V_H-S106		vdW
V_H-S108		vdW
V_H-C109		vdW
V_H-D57	K542	HB (sc-sc), SB
V_H-Y103		vdW
V_H-C104		vdW
V_H-S105		HB (mc-mc)
V_H-S106		HB (mc/sc-mc)
V_H-C109		vdW
V_H-K102	Y543	vdW
V_H-Y103		vdW
V_H-C104		S-π
V_H-C109		S-π
V_H-S52	E544	HB (sc-sc)
V_H-V53		vdW
V_H-G54		HB (mc-sc)
V_H-Y103		HB (mc-mc)
V_H-S105		HB (sc-sc)
V_H-Y103	L545	π-CH, vdW
V_H-N31	D546	HB (sc-sc)
V_H-Y103		vdW
V_H-Y103	K547	HB (sc-sc)

"HB", "SB", "vdW" stand for hydrogen bond, salt bridge, and van der Waals interaction, respectively. "mc" indicates the main chain mediated contacts, "sc" indicates the side chain mediated contacts. V_H and V_L indicate the variable heavy and light chains of 2G11.
