# Supplementary material for: A belt-buckle checkpoint regulates the onset of botulinum neurotoxin intoxication
Source: Nat Commun. 2026 Jun 23;17:5562. doi: 10.1038/s41467-026-74499-7 (PMC13291336; doi:10.1038/s41467-026-74499-7)
Supplement: Supplementary file 2 — Reporting Summary [file 41467_2026_74499_MOESM2_ESM.pdf]

nature portfolio

Corresponding author(s): Rongsheng Jin & Andreas Rummel

Last updated by author(s): May 28, 2026

Reporting Summary

Nature Portfolio wishes to improve the reproducibility of the work that we publish. This form provides structure for consistency and transparency in reporting. For further information on Nature Portfolio policies, see our Editorial Policies and the Editorial Policy Checklist.

Statistics

For all statistical analyses, confirm that the following items are present in the figure legend, table legend, main text, or Methods section.

n/a	Confirmed	
[ ]	[x]	The exact sample size (*n*) for each experimental group/condition, given as a discrete number and unit of measurement
[ ]	[x]	A statement on whether measurements were taken from distinct samples or whether the same sample was measured repeatedly
[ ]	[x]	The statistical test(s) used AND whether they are one- or two-sided<br>*Only common tests should be described solely by name; describe more complex techniques in the Methods section.*
[x]	[ ]	A description of all covariates tested
[x]	[ ]	A description of any assumptions or corrections, such as tests of normality and adjustment for multiple comparisons
[ ]	[x]	A full description of the statistical parameters including central tendency (e.g. means) or other basic estimates (e.g. regression coefficient) AND variation (e.g. standard deviation) or associated estimates of uncertainty (e.g. confidence intervals)
[ ]	[x]	For null hypothesis testing, the test statistic (e.g. *F*, *t*, *r*) with confidence intervals, effect sizes, degrees of freedom and *P* value noted<br>*Give P values as exact values whenever suitable.*
[x]	[ ]	For Bayesian analysis, information on the choice of priors and Markov chain Monte Carlo settings
[x]	[ ]	For hierarchical and complex designs, identification of the appropriate level for tests and full reporting of outcomes
[x]	[ ]	Estimates of effect sizes (e.g. Cohen's *d*, Pearson's *r*), indicating how they were calculated

Our web collection on statistics for biologists contains articles on many of the points above.

Software and code

Policy information about availability of computer code

Data collection	- Cryo-EM data were collected from Pacific Northwest Center for Cryo-EM.<br>- UNICORN v5.2 (https://www.cytivalifesciences.com/) was used to collect chromatography data in protein purification<br>- ImageJ for quantifying western blots
Data analysis	- Cryo-EM data were processed using cryoSPARC v4.4.1; Coot v.0.8.9.2 and Phenix v1.20 were used to build, refine and evaluate all the atomic models;<br>- PyMol 3.0.3 and ChimeraX were used to generate figures.<br>- GraphPad Prism v10.1.2 (https://www.graphpad.com/) and MS Excel were used for graphs.

For manuscripts utilizing custom algorithms or software that are central to the research but not yet described in published literature, software must be made available to editors and reviewers. We strongly encourage code deposition in a community repository (e.g. GitHub). See the Nature Portfolio guidelines for submitting code & software for further information.

Data

Policy information about availability of data

All manuscripts must include a data availability statement. This statement should provide the following information, where applicable:

- Accession codes, unique identifiers, or web links for publicly available datasets
- A description of any restrictions on data availability
- For clinical datasets or third party data, please ensure that the statement adheres to our policy

All source data are available within the paper.

Research involving human participants, their data, or biological material

Policy information about studies with human participants or human data. See also policy information about sex, gender (identity/presentation), and sexual orientation and race, ethnicity and racism.

Reporting on sex and gender	N/A
Reporting on race, ethnicity, or other socially relevant groupings	N/A
Population characteristics	N/A
Recruitment	N/A
Ethics oversight	N/A

Note that full information on the approval of the study protocol must also be provided in the manuscript.

Field-specific reporting

Please select the one below that is the best fit for your research. If you are not sure, read the appropriate sections before making your selection.

[x] Life sciences [ ] Behavioural & social sciences [ ] Ecological, evolutionary & environmental sciences

For a reference copy of the document with all sections, see nature.com/documents/nr-reporting-summary-flat.pdf

Life sciences study design

All studies must disclose on these points even when the disclosure is negative.

Sample size	N/A
Data exclusions	No data were excluded from the analysis.
Replication	ANS binding, P19N cleavage assay, MPN and MPN-BNA measurements and protein melting assays were replicated at least 3 times. All attempts at replication were successful.
Randomization	The experiments did not require randomization.
Blinding	The experiments did not require randomization.

Reporting for specific materials, systems and methods

We require information from authors about some types of materials, experimental systems and methods used in many studies. Here, indicate whether each material, system or method listed is relevant to your study. If you are not sure if a list item applies to your research, read the appropriate section before selecting a response.

Materials & experimental systems

n/a	Involved in the study
☐	☒ Antibodies
☐	☒ Eukaryotic cell lines
☒	☐ Palaeontology and archaeology
☐	☒ Animals and other organisms
☒	☐ Clinical data
☒	☐ Dual use research of concern
☒	☐ Plants

Methods

n/a	Involved in the study
☒	☐ ChIP-seq
☒	☐ Flow cytometry
☒	☐ MRI-based neuroimaging

Antibodies

Antibodies used

Anti-BoNT/A1 monoclonal antibody (2G11) obtained from James Marks' lab.
BAT: US FDA-licensed equine heptavalent botulism antitoxin
Cleaved SNAP-25 was detected by the mouse monoclonal anti-SNAP-25 antibody clone 71.2 (#111111; Synaptic Systems GmbH, Göttingen, Germany) while VAMP-2 serving as control protein was detected by the mouse monoclonal anti-VAMP-2 antibody clone 69.1 (#104211; Synaptic Systems GmbH, Göttingen, Germany).

Validation

The validation of 2G11 is described in two previous published papers:
1. Levy R, Forsyth CM, LaPorte SL, Geren IN, Smith LA, Marks JD. Fine and domain-level epitope mapping of botulinum neurotoxin type A neutralizing antibodies by yeast surface display. J Mol Biol. 2007 Jan 5;365(1):196-210. doi: 10.1016/j.jmb.2006.09.084. Epub 2006 Oct 3. PMID: 17059824; PMCID: PMC1994578.
2. Lou J, Geren I, Garcia-Rodriguez C, Forsyth CM, Wen W, Knopp K, Brown J, Smith T, Smith LA, Marks JD. Affinity maturation of human botulinum neurotoxin antibodies by light chain shuffling via yeast mating. Protein Eng Des Sel. 2010 Apr;23(4):311-9. doi: 10.1093/protein/gzq001. Epub 2010 Feb 15. PMID: 20156888; PMCID: PMC2841544.

Eukaryotic cell lines

Policy information about cell lines and Sex and Gender in Research

Cell line source(s)

Cell line: P-19
DSMZ (German cell bank) no.: ACC 316
Species: murine (mouse) (Mus musculus)
Cell type: embryonal carcinoma
https://webshop.dsmz.de/en/human-animal-cell-lines/P-19.html

Authentication

Species-level identification was carried out by mitochondrial Cytochrome C Oxidase Subunit 1 (COI) DNA barcoding according standard ANSI/ATCC ASN-0003-2015 and revealed Mus musculus species.

Mycoplasma contamination

negative in DAPI, microbiological culture, RNA hybridization, PCR assays

Commonly misidentified lines
(See ICLAC register)

Name any commonly misidentified cell lines used in the study and provide a rationale for their use.

Animals and other research organisms

Policy information about studies involving animals; ARRIVE guidelines recommended for reporting animal research, and Sex and Gender in Research

Laboratory animals

Mus musculus, RjOrl:SWISS (CD-1®)

Wild animals

Provide details on animals observed in or captured in the field; report species and age where possible. Describe how animals were caught and transported and what happened to captive animals after the study (if killed, explain why and describe method; if released, say where and when) OR state that the study did not involve wild animals.

Reporting on sex

female, but no sex-dependence observed in previous in-house studies

Field-collected samples

For laboratory work with field-collected samples, describe all relevant parameters such as housing, maintenance, temperature, photoperiod and end-of-experiment protocol OR state that the study did not involve samples collected from the field.

Ethics oversight

The use of tissue of mice in the MPN assay is by law not an animal experiment according to §4 Abs. 3 (killing of animals for scientific purposes, German animal protection law (TSchG)). The use of the mice was notified as project license no. 2018/209 to the animal welfare officer of the Central Animal Laboratory of Hannover Medical School. Consumed animal numbers are reported annually to the Central Animal Laboratory of Hannover Medical School and to the respective authority Veterinäramt Hannover and LAVES.

Note that full information on the approval of the study protocol must also be provided in the manuscript.

Plants

Seed stocks	*Report on the source of all seed stocks or other plant material used. If applicable, state the seed stock centre and catalogue number. If plant specimens were collected from the field, describe the collection location, date and sampling procedures.*
Novel plant genotypes	*Describe the methods by which all novel plant genotypes were produced. This includes those generated by transgenic approaches, gene editing, chemical/radiation-based mutagenesis and hybridization. For transgenic lines, describe the transformation method, the number of independent lines analyzed and the generation upon which experiments were performed. For gene-edited lines, describe the editor used, the endogenous sequence targeted for editing, the targeting guide RNA sequence (if applicable) and how the editor was applied.*
Authentication	*Describe any authentication procedures for each seed stock used or novel genotype generated. Describe any experiments used to assess the effect of a mutation and, where applicable, how potential secondary effects (e.g. second site T-DNA insertions, mosiacism, off-target gene editing) were examined.*
