# Supplementary material for: A belt-buckle checkpoint regulates the onset of botulinum neurotoxin intoxication
Source: Nat Commun. 2026 Jun 23;17:5562. doi: 10.1038/s41467-026-74499-7 (PMC13291336; doi:10.1038/s41467-026-74499-7)
Supplement: Supplementary file 3 — Transparent Peer Review file [file 41467_2026_74499_MOESM3_ESM.pdf]

nature portfolio

Peer Review File

A belt-buckle checkpoint regulates the onset of botulinum neurotoxin intoxication

Corresponding Author: Dr Rongsheng Jin

This file contains all reviewer reports in order by version, followed by all author rebuttals in order by version.

Version 0:

Reviewer comments:

Reviewer #1

(Remarks to the Author)
The manuscript by Chen et al., “Modulating the belt of botulinum neurotoxin to control the onset of intoxication” explores the properties of the belt region in the botulinum toxins, using BoNT/A1 and BoNT/E as models. The authors show that the flexibility of region of the protein controls the onset of intoxication - faster BoNT/E LC domain delivery to the cytosol and cleavage of the SNARE proteins correlates with the flexibility of the belt. The authors also describe a motif they term “belt-buckle” - loosening this motif makes the protein more sensitive to lower pH and capable of faster translocation into the cytosol / SNARE cleavage. Antibody binding to this region neutralises BoNT/A.

The manuscript is very well written and the experiments are well designed and executed.

I have two major comments that the authors should improve on:

1. The authors need to provide more convincing density map illustrations, validating their models for the key elements of the structure they are talking about. Currently it is not easy to judge the quality of the densities corresponding to the belt buckle and the belt in the different structures - it would be useful to have a figure (or figures) showing more clearly the quality of the densities of interest and the corresponding models. To what extent can the authors reliably model the models the way they did?

2. The obvious missing bit in the manuscript is the cryo-EM analysis of the BoNT/A1 WFY mutant. What happens to this mutant, with or without the antibody? The authors make compelling biochemical arguments, but the cryo-EM analysis of this mutant would be a powerful addition to this story, showing directly what the molecular effects of WFY mutations are.

I also have a few other points, which I hope will help the authors in improving their manuscript:

Abstract. “promotes quicker LC unfolding” - is there evidence in this manuscript that LC unfolds? It may be safer to just state that LC translocates.

Line 58. “how BoNT/E responds”, not responses.

Figure 1b and d - I find it a bit difficult to relate these two figure panels, since two parameters of model presentation have been varied (the surface was changed to ribbons, and the view angle was also flipped). Why not at least keep the same angle for b and d? Or perhaps add the surface representation to go along with the same view as in d. This is exemplified nicely in Figure 2a-b, where the same views are used, which makes identification of the image features a lot easier.

Fig 1d appears somewhat 2-dimensional, because the ribbons are rather thin and the depth cue is not strong. Adding a stronger depth cue would help get rid of this effect (possibly).

Figure 2e - close or closed?

The quality of the supplementary figures is not great - if these are made in Pymol, please render the images. The current quality is very poor - pixelated images of structures (especially Supp Figure 1), too small to see anything.

Supplementary Figure 2, 5 - the raw micrographs are too small, pixelated and include circles that obscure the image. Please improve this.

Supplementary Figure 4c. The labelling with “h”, “sec” on the left and right of the blots is somewhat confusing. Can the authors optimize the labelling / arrangement of this figure panel so that it is more clear what is being shown? At the moment the labels are positioned in rather complicated way, which makes the figure difficult to read.

Reviewer #2

(Remarks to the Author)
Botulinum neurotoxin (BoNT) is the most toxic protein known, yet it is also widely used for medical and cosmetic purposes. BoNT/A exhibits a slower onset of action compared with BoNT/E. The authors propose that the “belt,” a long loop wrapping around the catalytic LC domain, is more flexible in BoNT/E than in BoNT/A, and the enhanced flexibility allows faster onset of action of BoNT/E. They further show that cryo-EM structures of BoNT/E under acidic conditions reveal an ensemble of open conformations. The diversity of these conformations suggests enhanced belt flexibility in BoNT/E at low pH.
Next, the authors engineered the belt of BoNT/A to weaken its interaction with LC–HN, which resulted in a faster onset of action. They also identified mutations in the “belt buckle” that increase susceptibility to acidic pH. This BoNT/A mutant displayed faster onset of action in neuronal cell culture. In addition, they identified the antibody 2G11, which recognizes the belt buckle of BoNT/A. Cryo-EM analysis revealed that 2G11 locks the belt buckle, thereby restricting conformational changes under acidic conditions.
The structural data are of high quality, and the biochemical assays appear to have been carefully performed. This manuscript consists of the BoNT/E part and the BoNT/A part. In my opinion, these two parts are not sufficiently connected to justify being presented together in a single paper. In addition, I wonder why the authors did not analyze the BoNT/A-WFY by cryo-EM. My specific concerns and comments are as follows and should be addressed.

1.The authors state that the belt of BoNT/E is more flexible than that of BoNT/A, based on structural comparisons. However, since the overall conformations of BoNT/E and BoNT/A differ substantially, such a comparison may not directly reflect the intrinsic flexibility of the belt. In BoNT/E, the belt appears more flexible around the middle region due to the lack of interaction with the LC-HN domain. Nevertheless, the authors were unable to identify an effective mutation in the corresponding region of BoNT/A that increases belt flexibility. If the flexibility of the belts of the two toxins is to be discussed in the same context, this discrepancy should be clarified.

2.The authors did not attempt to engineer mutant BoNT/E to stabilize the belt, citing concerns about potential artifacts arising from non-specific interfering effects of mutagenesis. However, since the title includes the phrase “Modulating the belt … to control the onset of intoxication,” it would strengthen the study to test whether stabilizing the belt could indeed alter the onset of action, either in BoNT/A or BoNT/E. For example, did the authors consider introducing a mutation in the belt buckle of BoNT/E to enhance its interaction and mimic the more stable conformation observed in BoNT/A?

3.The cryo-EM structures of BoNT/E at acidic pH reveal an ensemble of open conformations, including both open-wing and semi-closed states. It would be helpful if the authors could explain why these open conformations are favored at low pH. The manuscript suggests that the HCE moves away from the rigid LC–HN under acidic conditions; is this movement related to the enhanced flexibility of the belt? Could the interaction between HC and the belt also be weakened at low pH? In addition, a comparison with the recent cryo-EM structure of BoNT/A bound to SV2B at acidic pH, in which the HN domain is positioned close to the membrane, would provide useful context. As full-length SV2A/SV2B structures are now available, the authors might also consider modeling the complex structure of the open conformation of BoNT/E with SV2A/B.

4. The author states that the BoNT/A-WFY mutant loosens the belt without affecting other parts of the toxin. Since the belt-buckle region plays an important role in anchoring the LC and HN, I am concerned about potential artificial effects caused by this mutation. For instance, is the disulfide bond in the belt-buckle maintained? Additionally, why is the SEC peak of BoNT/A-WFY slightly shifted compared to that of the wild-type (Fig. 4a)? I recommend that the author provide structural analysis of BoNT/A-WFY using cryo-EM.

5. The BoNT/A-WFY exhibits only ~40% of the wild-type activity in the MPN assay, whereas it shows more robust activity in the cleavage assay using neuronal cell culture. Please clarify this discrepancy. In addition, while the authors generally refer to BoNT/A, Supplementary Figure 4a specifies “engineered BoNT/A,” which contains a short peptide including a thrombin site for additional cleavage. This should be described in the main text, along with an explanation of why this mutant protein was used.

6. The authors conclude that the 2G11 antibody, which recognizes the belt-buckle, neutralizes BoNT/A by inhibiting the conformational change induced at acidic pH. However, since the HC domain moves flexibly relative to the LC-HN in the cryo-EM structure, the antibody does not appear to inhibit HC movement. Could the authors provide experimental evidence demonstrating that the antibody blocks the conformational transition?

Minor
1. The authors conclude that the belt of BoNT/E has greater flexibility than that of BoNT/A, based on comparisons with previously reported crystal structures. It would be valuable for the authors to also discuss the reported cryo-EM structures of BoNT/E and BoNT/A (bound to SV2B), which may provide further insights into belt flexibility.

2. Please clarify how the percentage of cleavage activity was calculated from the Western blots in the P19 SNAP-25 assay. In particular, it would be helpful to specify the quantification workflow (band selection for full-length vs. cleaved species, background subtraction, and normalization to a loading control or total protein), confirm that signals were within the linear range, and provide the exact formula used.

3. Line 89, “corresponding to the N-terminal segment of the HC…” This sentence should be revised for clarity.

4. Line 438, please confirm whether “magnification of 29K” is correct?

5. Could the authors clarify how the sequence of 2G11 was determined? If possible, providing the amino acid sequence would enhance the manuscript’s completeness.

6. Lines 266–275: This section seems somewhat confusing, and the manuscript may read more clearly if it is omitted.

7. Line 761: The description of the black arrows is unclear. It may be more informative to indicate the specific residues contributing to the interaction instead.

8. Figure 2d, Does “BoNT/E (open)” refer to “BoNT/E (M-PTC form)”?

9. Related to Figure 3f, the melting curve must be provided in the Supplementary.

10. In Supplementary Figure 2 and 5, higher resolution figures must be provided. Especially, the micrographs are of poor quality.

Reviewer #3

(Remarks to the Author)
I believe that the evidence presented by this study is rigorous and robust. By direct molecular (mutation of the belt segment) and pharmacological (antibody binding) interventions, serving to respectively increase or reduce the fluidity (tightness) of the belt conformation they are able to support the hypothesis that the rapidity of the time course of the onset of BoNtE. as compared to BoNtA reflects the differences in the degree to which the belt structure that encompases the BoNtA light chain constrains the rate of unfolding of the light chain to achieve the active cleaving conformation.

The case is made that this principle has evident clinical utility as it permits a better control of the therapeutic effects arising from local block of terminal release (particular the cholinergic terminal).
It is additionally clear that the role of LC belt points to the critical role played by stabilizing the belt in addressing issues of toxicity.

I have only a few comments.

While Both of BoNT/A and BoNT/E. employ the synaptic vesicle glycoprotein 2 (SV2) as the receptor, there are three paralogs of SV2s (A,B,C). It is my understanding that the respective affinity by BoNtA and E differ with the former showing the highest affinity for SV2C and the later employing, SV2A,B. and not C. I am not clear on the differential distribution of the paralogs in the cholinergic terminal (e.g. relevant to the hemidiaphram). Please address the speculation that the differential onset of block might reflect that all three paralogs are in the cholinergic terminal and the onset reflects a more a more rapid uptake cleavage is not due to the role of SV2A/B for BoNtE and only on SV2C for BoNtA.

It is clear to me that the time course of unfolding of the BoNT construct is regulated by the tightness of the belt and that where the target effect is local (as in delivery to the cutaneous cholinergic terminal), changing the belt tightness can enhance the unfolding revealing the active form. Here the time course. largely depends upon the time of uptake and then upon time of onset of the consequence of the cleavage.

There is, however, considerable evidence that many effects of several isoforms of BoNT may be mediated by its uptake in the terminals and transport to the cell bodies. Such BoNT transport has been well documented. Models focusing on the time to onset of effects upon pain behavior (PMID: 39196607; PMID: 38452215.; PMID: 25958249) and the earlier effects upon plasma (PMID: 24333775are consistent with the cleavage of SNAREs in the terminals and then the DRG.

In that I regard, I note that BoNT/A and BoNT/E has been reported to be. internalized in non-acidic axonal carriers and undergo fast axonal retrograde transport in motor neurons. BoNT A reportedly at rates of 0.8 µm/s (PMID: 23300443). I do not know such data on BoNT E transport velocities and while it is not likely to be that much more more rapidly transported than the BoNtA, it still reflects upon the potential role of transport velocity n defining the time of effect onset.

Would you care to speculate on the role of belt tightness on such ortho /antidromic transport and perhaps note that some clinically relevant delays may reflect time to affect the central terminals.

Several references
Marino MJ, Terashima T, Steinauer JJ, Eddinger KA, Yaksh TL, Xu Q. Botulinum toxin B in the sensory afferent: transmitter release, spinal activation, and pain behavior. Pain. 2014 Apr;155(4):674-684. doi: 10.1016/j.pain.2013.12.009. Epub 2013

Dec 11. PMID: 24333775; PMCID: PMC3960322.

Moreau N, Korai SA, Sepe G, Panetsos F, Papa M, Cirillo G. Peripheral and central neurobiological effects of botulinum toxin A (BoNT/A) in neuropathic pain: a systematic review. Pain. 2024 Aug 1;165(8):1674-1688. doi: 10.1097/j.pain.0000000000003204. Epub 2024 Mar 5. PMID: 38452215.

Ramachandran R, Lam C, Yaksh TL. Botulinum toxin in migraine: Role of transport in trigemino-somatic and trigemino-vascular afferents. Neurobiol Dis. 2015 Jul;79:111-22. doi: 10.1016/j.nbd.2015.04.011. Epub 2015 May 6. PMID: 25958249; PMCID: PMC4458441.

Restani L, Giribaldi F, Manich M, Bercsenyi K, Menendez G, Rossetto O, Caleo M, Schiavo G. Botulinum neurotoxins A and E undergo retrograde axonal transport in primary motor neurons. PLoS Pathog. 2012 Dec;8(12):e1003087. doi: 10.1371/journal.ppat.1003087. Epub 2012 Dec 27. PMID: 23300443; PMCID: PMC3531519.

Yeo JC, Tay FP, Bennion R, Loss O, Maignel J, Pons L, Foster K, Beard M, Bard F. Botulinum toxin intoxication requires retrograde transport and membrane translocation at the ER in RenVM neurons. Elife. 2024 Aug 28;12:RP92806. doi: 10.7554/eLife.92806. PMID: 39196607; PMCID: PMC11357346.

Version 1:

Reviewer comments:

Reviewer #1

(Remarks to the Author)
I think the submitted manuscript is greatly improved, but it requires additional work. Although the authors have adequately addressed most of the comments, the new structure of BoNT/Ai-WFY is deeply problematic. There is basically no structure - just an overfitted 3D volume with essentially no protein-like features. The supplied density map is a collection of loosely connected hollow volumes and there is no way this can be published in the current form. What the authors have at the moment is not a cryo-EM structure at 5Å resolution but a hallucination.

Because of this, the manuscript requires careful reevaluation. The new structural data on the mutant must be presented in a different way, and I think it is ok to realise that this mutant may not produce a high resolution structure. If the cryo-EM imaging of WFY is to be kept in the manuscript, the corresponding cryo-EM analysis must be at least conclusive.

The authors might want to consider continuing 3D classification to try and extract a better 3D volume from their current one (301,287 particles is a large dataset). Whether this will produce any results is currently hard to judge, but it's worth trying.

The local resolution-coloured map in its current form is basically meaningless (Supplementary Figure 5), because the volume that was produced by non-uniform refinement lacks any clear features.

The authors wrote "The overall structure of BoNT/Ai-WFY is almost identical to the crystal structure of WT BoNT/A (PDB: 3BTA) (new Supplementary Fig. 5-6, next page)." in the rebuttal. Even with the most charitable approach to the available "3D reconstruction" of BoNT/Ai-WFY, the fit of the 3BTA is very poor. The HN domain matches to a region where there is no density. The extra bit of density in the bottom of the blobby map in Supplementary Figure 6 does not match the HN domain (or any other domain of the toxin). Regardless of all this, it is not worth spending any time analysing the fit of an X-ray structure to an overfitted 3D volume that does not correspond to the protein of interest.

Given the current lack of any conclusive results on cryo-EM analysis of the WFY mutant, that whole part of the manuscript needs to be reworked and supplementary figures 5-6 need to be modified accordingly. It is possible that there will not be a structure (even at low resolution) despite the best efforts of the authors. They should consider removing this part from the manuscript, should they reach this conclusion.

Reviewer #2

(Remarks to the Author)
I appreciate the authors' faithful efforts in addressing the previous comments. The newly presented cryo-EM structure of BoNT/Ai-WFY is reported at approximately 5 Å resolution; however, even secondary structure elements do not appear to be well resolved (Supplementary Figure 6). While I understand the authors' interpretation that this may be due to increased structural flexibility, the current cryo-EM map appears more indicative of overall structural destabilization rather than localized domain movement. At the very least, it is not entirely convincing to conclude that the overall structure is nearly identical to that of the crystal structure of the wild-type protein (Page 6). Could the authors clarify whether additional efforts were made to improve the map quality, such as local refinement or more extensive 3D classification? The authors report selecting approximately ~30 k particles as the best class from an initial ~400k particles. It would be helpful to know whether other classes were also evaluated and whether any of them yielded maps with improved resolution.

The BoNT/As used in the activity assays are mutant proteins introducing thrombin cleavage sites. It is unclear whether such mutants are commonly used for activity assays in this field. Although the authors added the explanation about this in the methods in the revised manuscript accroding to the reviewer's comment, this point should be clarified in the main text (page 6, around MPN assay), along with an explanation of how the introduced mutations may affect the activity and interpretation of the results.

I still have concerns regarding the reported magnification of 29k×. The dataset for BoNT/Ai-2G11 was collected at 105k× magnification with a pixel size of 0.8266 Å, whereas the dataset for either LC-HN/E or BoNT/Ei was collected at 29k× with a pixel size of 0.788 Å. As both datasets were acquired using the same microscope at the same facility, the smaller pixel size for the latter case would typically correspond to a higher nominal magnification than 105,000× of the former case. Could the authors please verify whether the reported magnification and pixel size values are correct?

I found several typos. For example, “Thermal Fisher Vitrobot M” in the last sentence on Page 12 should be “Thermofisher Vitrobot Mark IV”. Please proofread the entire manuscript carefully.

Reviewer #3

(Remarks to the Author)
The response to my review is appropriate and adequate. My view of the potential contribution of transport to the to the time of onset issue while not relevant to the results of the in vitro/ex vivo results characterizing the role of the belt are potentially relevant to the issue of time of onset for event that almost certainly reflect upon a central effect of BoNTs delivered to the peripheral terminal (e.g as in the use in managing pain of a neuropathic origin and in migraine.

Tony L. Yaksh

Version 2:

Reviewer comments:

Reviewer #1

(Remarks to the Author)
I am overall satisfied with the revised manuscript. I think the authors will have to accept the fact that the WFY mutant is substantially perturbed, even if the negative stain suggests that similar shapes can be observed for this mutant as for the wild-type. I am not sure the wording they choose in the description of the negative stain results is optimal, but I leave it to the authors to decide how to deal with this.

It is clear that the construct is badly affected, so a statement like "No obvious large-scale conformational differences were observed between BoNT/Ai-WT and BoNT/Ai-WFY at the negative-stain level, suggesting that the WFY mutations do not substantially perturb the overall architecture of the toxin" can be misread / misinterpreted by the reader, even if a disclaimer follows in the following sentences. In my opinion it would be safer to avoid the words "conformational" (the conformation of WFY clearly is very much affected, and the negative stain data hardly substitute cryo-EM analysis in this case) and "architecture" (this word is not defined here, and given the clear disruption of the structure this is just confusing). The 2D classes of the WFY in negative stain are similar, but not the same as those of the wild-type, so the statement about the large-scale conformational differences is probably not quite right.

There must be a more straightforward way to state that the WFY mutant structure is perturbed / destabilized, even though negative staining of the sample indicates that the gross features of the particles appear to be preserved.

Reviewer #2

(Remarks to the Author)
I have no further comments and recommend this manuscript for publication.

Open Access This Peer Review File is licensed under a Creative Commons Attribution 4.0 International License, which permits use, sharing, adaptation, distribution and reproduction in any medium or format, as long as you give appropriate credit to the original author(s) and the source, provide a link to the Creative Commons license, and indicate if changes were made.
In cases where reviewers are anonymous, credit should be given to 'Anonymous Referee' and the source.
The images or other third party material in this Peer Review File are included in the article's Creative Commons license, unless indicated otherwise in a credit line to the material. If material is not included in the article's Creative Commons license and your intended use is not permitted by statutory regulation or exceeds the permitted use, you will need to obtain permission directly from the copyright holder.
To view a copy of this license, visit https://creativecommons.org/licenses/by/4.0/

Point-by-point Response

We thank the reviewers for their collective efforts in reviewing our manuscript, their positive view of our work, and their constructive comments to improve our manuscript. As detailed below, we have addressed all the critiques raised and incorporated them into the revised manuscript.

Response to Reviewer 1

1. The authors need to provide more convincing density map illustrations, validating their models for the key elements of the structure they are talking about. Currently it is not easy to judge the quality of the densities corresponding to the belt buckle and the belt in the different structures - it would be useful to have a figure (or figures) showing more clearly the quality of the densities of interest and the corresponding models. To what extent can the authors reliably model the models the way they did?

Response:

We have modified Figure S1 to better illustrate the density of the belt regions. Instead of only showing the backbone structures in the original version, we now show all the side chains of amino acids on the belt. Most of the side chains in the visible portion of the belt in our BoNT/E cryo-EM structure (panels d and e) are well resolved, demonstrating the high quality of the EM map and the reliability of our model. Please note that the electron densities shown in panels a-c were calculated using the previously published structures.

2. The obvious missing bit in the manuscript is the cryo-EM analysis of the BoNT/A1 WFY mutant. What happens to this mutant, with or without the antibody? The authors make compelling biochemical arguments, but the cryo-EM analysis of this mutant would be a powerful addition to this story, showing directly what the molecular effects of WFY mutations are.

Response:

We thank the reviewer for this suggestion. We have devoted significant effort to resolve the cryo-EM structure of BoNT/Ai-WFY at 4.95 Å resolution. The moderate resolution of this structure was likely restricted by the loosening of the belt and the associated increase in structural flexibility. The overall structure of BoNT/Ai-WFY is almost identical to the crystal structure of WT BoNT/A (PDB: 3BTA) (new Supplementary Fig. 5-6, next page). Structural modeling revealed that LC/A and H_CA domains remain intact, suggesting their activities are not affected by the WFY mutations. Nevertheless, the first segment of the belt (i.e. residues F471–N493), a portion of H_NA that interacts with this part of the belt (i.e. residues P669–W717), and the belt-buckle did not have visible densities, indicating high structural flexibility in these regions (new Supplementary Fig. 6). These structural findings confirm that weakening the belt-buckle by the WYF mutations enhances the flexibility of the belt of BoNT/A. Interestingly, a portion of H_NA that directly interacts with the belt also becomes more flexible, which is likely due to the loss of mutual support between H_NA and the belt in this region. It suggests that weakening the belt-buckle could also affect the structure and function of the H_N, and thus the dynamics of LC translocation. These new findings are now included in the revised manuscript.

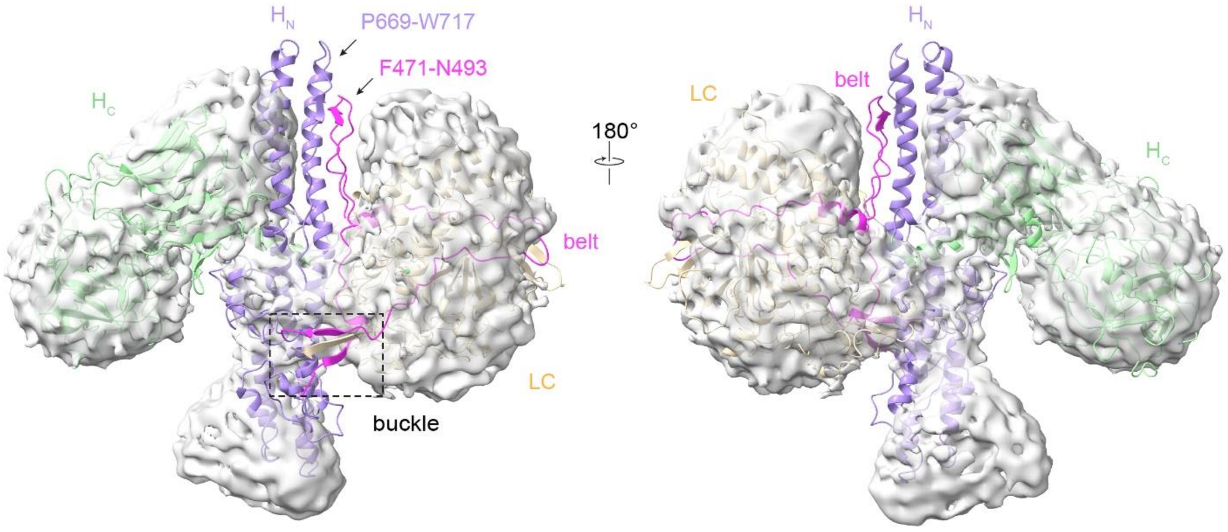

Supplementary Figure 6. Structural characterization of the engineered BoNT/Ai-WFY. Fitting the structure of the WT BoNT/A (PDB: 3BTA, LC: wheat, H_N: purple, H_C: pale green, Belt: hot pink) into the cryo-EM density of BoNT/Ai-WFY resolved at 4.95 Å resolution. The moderate resolution of this structure was likely restricted by the loosening of the belt and the associated increase in structural flexibility. Structural modeling shows that the first segment of the belt (residues F471–N493), a portion of H_NA (residues P669–W717), and the belt-buckle did not have visible densities, indicating high structural flexibility in these regions.

Abstract. "promotes quicker LC unfolding" - is there evidence in this manuscript that LC unfolds? It may be safer to just state that LC translocates.

Response: We made the change as suggested.

Line 58. "how BoNT/E responds", not responses.

Response: Corrected.

Figure 1b and d - I find it a bit difficult to relate these two figure panels, since two parameters of model presentation have been varied (the surface was changed to ribbons, and the view angle was also flipped). Why not at least keep the same angle for b and d? Or perhaps add the surface representation to go along with the same view as in d. This is exemplified nicely in Figure 2a-b, where the same views are used, which makes identification of the image features a lot easier.

Fig 1d appears somewhat 2-dimensional, because the ribbons are rather thin and the depth cue is not strong. Adding a stronger depth cue would help get rid of this effect (possibly).

Response:

Figure 1d was modified as suggested. The structure of BoNT/E is consistently shown as surface model in panel 1b and 1d. The reason for a 180° rotation between the two panels is to show both the front and the back of this structure. The belt-buckle can be clearly seen in panel 1b,

while the region of the belt that is sandwiched between LC/E and H_CE (the boxed region enlarged in panel 1e) can be better seen in panel 1d.

Figure 2e - close or closed?

Response: It should be closed. Corrected.

The quality of the supplementary figures is not great - if these are made in Pymol, please render the images. The current quality is very poor - pixelated images of structures (especially Supp Figure 1), too small to see anything.

Response:

The low quality of the supplementary figures was partly due to the improper conversion from the Word format to PDF. In addition, we have remade Supplementary Figures 1, 2, 4 and 7 to improve clarity.

Supplementary Figure 2, 5 - the raw micrographs are too small, pixelated and include circles that obscure the image. Please improve this.

Response:

We remade Supplementary Fig. 2 and Fig. 5 (now Fig. S7) to show bigger and clearer micrographs and reorganized the panels to improve clarity.

Supplementary Figure 4c. The labelling with “h”, “sec” on the left and right of the blots is somewhat confusing. Can the authors optimize the labelling / arrangement of this figure panel so that it is more clear what is being shown? At the moment the labels are positioned in rather complicated way, which makes the figure difficult to read.

Response:

We improved the labelling and arrangement of this figure, which now clearly shows the toxin incubation time and the Western blots exposure time.

Response to Reviewer 2

The structural data are of high quality, and the biochemical assays appear to have been carefully performed. This manuscript consists of the BoNT/E part and the BoNT/A part. In my opinion, these two parts are not sufficiently connected to justify being presented together in a single paper. In addition, I wonder why the authors did not analyze the BoNT/A-WFY by cryo-EM. My specific concerns and comments are as follows and should be addressed.

Response:

We agreed with the reviewer that this manuscript seems to have two parts on BoNT/E and BoNT/A, respectively. However, they represented two stages of a cohesive story: structural studies of BoNT/E suggested a role for the belt in regulating the onset of action, a hypothesis

subsequently validated by our ‘reverse-engineering’ studies of BoNT/A. We cannot exclude the possibility that one could attempt to validate this hypothesis by engineering BoNT/E to stabilize the belt and slow down its action. However, such an approach is highly susceptible to artifacts arising from non-specific interference. In addition, a BoNT/E with a delayed onset of action is of little pharmaceutical interest. In contrast, the identification of a fast-acting variant of BoNT/A not only validates the BoNT/E-derived hypothesis, but also has tremendous translational value. We have determined the cryo-EM structure of BoNT/Ai-WFY during the revision. Please see our response to Reviewer-1 for details.

1. The authors state that the belt of BoNT/E is more flexible than that of BoNT/A, based on structural comparisons. However, since the overall conformations of BoNT/E and BoNT/A differ substantially, such a comparison may not directly reflect the intrinsic flexibility of the belt. In BoNT/E, the belt appears more flexible around the middle region due to the lack of interaction with the LC-HN domain. Nevertheless, the authors were unable to identify an effective mutation in the corresponding region of BoNT/A that increases belt flexibility. If the flexibility of the belts of the two toxins is to be discussed in the same context, this discrepancy should be clarified.

Response:

We agree with the reviewer that the belt flexibilities of BoNT/A and BoNT/E are different, which are affected by their amino acid compositions and their interactions with the rest of the toxin. It is possible that the mutations we introduced into the middle region of the BoNT/A belt were insufficient to induce substantial structural changes, or our biochemical assays were not sensitive enough to detect subtle effects. More comprehensive mutagenesis screening throughout the belt in follow-up studies may provide useful insights to further refine the design of fast-acting BoNT/A variants.

2. The authors did not attempt to engineer mutant BoNT/E to stabilize the belt, citing concerns about potential artifacts arising from non-specific interfering effects of mutagenesis. However, since the title includes the phrase “Modulating the belt … to control the onset of intoxication,” it would strengthen the study to test whether stabilizing the belt could indeed alter the onset of action, either in BoNT/A or BoNT/E. For example, did the authors consider introducing a mutation in the belt buckle of BoNT/E to enhance its interaction and mimic the more stable conformation observed in BoNT/A?

Response:

It is technically possible to “introducing a mutation in the belt buckle of BoNT/E to enhance its interaction and mimic the more stable conformation observed in BoNT/A”. However, even if such a mutant were to show a slower onset of action, interpreting this type of “negative” result would be challenging. Any mutation that perturbs the structure or function of BoNT/E—even outside the belt region—can nonspecifically reduce its activity, making it difficult to distinguish a mechanistic effect from general loss of activity. As a result, this approach would require substantial effort and resources to reach a meaningful conclusion. For this reason, we adopted the opposite strategy and engineered BoNT/A instead. In this context, a successful validation in BoNT/A of the BoNT/E-derived hypothesis will yield a positive outcome that directly supports the mechanistic model and effectively rules out non-specific artifacts.

While the direct molecular interventions (mutation of the belt segment) are not practical in this case, we instead explored pharmacological interventions (antibody binding) to “test whether

stabilizing the belt could indeed alter the onset of action, either in BoNT/A or BoNT/E". Specifically, we demonstrated that antibody 2G11 and a VHH (JLE-E9) stabilizes the belt-buckle of BoNT/A and BoNT/E, respectively, leading to toxin neutralization.

3. The cryo-EM structures of BoNT/E at acidic pH reveal an ensemble of open conformations, including both open-wing and semi-closed states. It would be helpful if the authors could explain why these open conformations are favored at low pH. The manuscript suggests that the HCE moves away from the rigid LC–HN under acidic conditions; is this movement related to the enhanced flexibility of the belt? Could the interaction between HC and the belt also be weakened at low pH? In addition, a comparison with the recent cryo-EM structure of BoNT/A bound to SV2B at acidic pH, in which the HN domain is positioned close to the membrane, would provide useful context. As full-length SV2A/SV2B structures are now available, the authors might also consider modeling the complex structure of the open conformation of BoNT/E with SV2A/B.

Response:

The transition into the open conformations of BoNT/E at acidic pH is expected to be mediated by the helical linker region connecting H_CE and H_NE, which is also affected by interactions between H_CE and the LC-H_N moiety. But the structural elements on BoNT/E that sense the pH change and trigger these conformational changes remain elusive. In similar scenario, Kammerer and colleagues observed pH-dependent as well as SV2B-binding dependent conformational changes of BoNT/A (ref. 26), but the pH sensing mechanism was also not identified in those cases.

As the reviewer pointed out, there have been at least seven papers about the full-length structures of SV2A/2B published recently, and some of them are cited in our manuscript. Two of these structures involve H_CA, one with BoNT/A, one with TeNT, and three with SV2 only. We modeled the complex structures of the closed and the open conformations of BoNT/E with SV2A (see below), but this analysis did not yield findings directly relevant to the scope of the current manuscript. To maintain coherence and focus, we chose not to include these results in the revised manuscript. Nevertheless, these recent papers provide extensive insights into the structures of SV2 isoforms, the structural flexibility of the luminal domain loop4, and the interactions between SV2 and BoNT/A or TeNT under different pH and functional states. Inspired by these developments, we plan to prepare a mini-review that systematically analyzes these structures and may reveal new insights and directions for future studies.

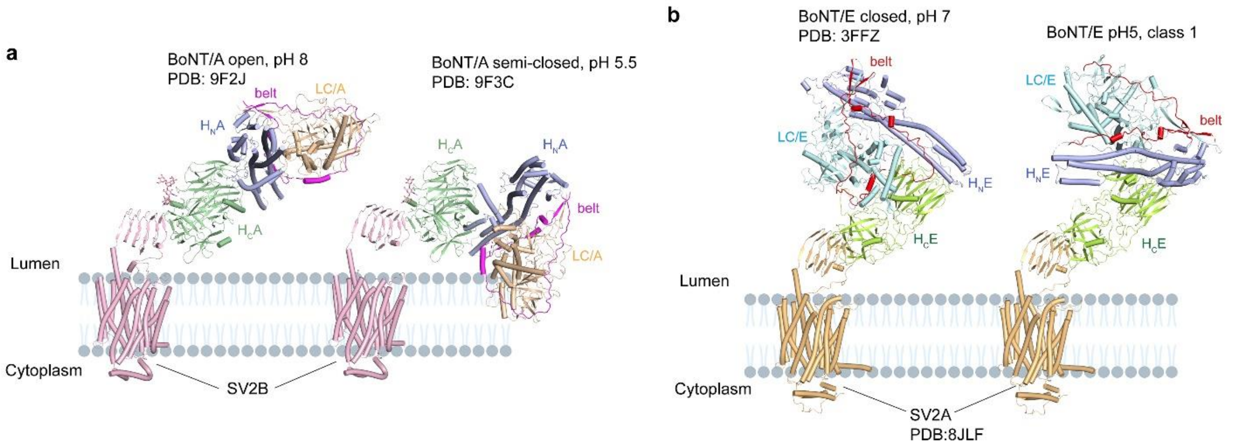

Structures of the BoNT/A–SV2B complexes and the modeled BoNT/E–SV2A complexes at different pH.

a. BoNT/A (open conformation, pH 8) in complex with SV2B (left panel). BoNT/A (semi-closed conformation, pH 5.5) in complex with SV2B (right panel).

b. Mode of BoNT/E (closed conformation, pH 7) in complex with SV2A (left panel). Mode of BoNT/E (class 1 conformation, pH 5, this study) in complex with SV2A.

4. The author states that the BoNT/A-WFY mutant loosens the belt without affecting other parts of the toxin. Since the belt-buckle region plays an important role in anchoring the LC and HN, I am concerned about potential artificial effects caused by this mutation. For instance, is the disulfide bond in the belt-buckle maintained? Additionally, why is the SEC peak of BoNT/A-WFY slightly shifted compared to that of the wild-type (Fig. 4a)? I recommend that the author provide structural analysis of BoNT/A-WFY using cryo-EM.

Response:

We conducted thrombin cleavage on LC-H_N/A, both the WT and the WFY mutant, each containing a thrombin cleavage site inserted between LC/A and H_N/A (Supplementary Figure 4a). The cleavage was complete, as only the cleaved LC/A and H_N/A were observed on the reducing gel that migrated separately. In contrast, these two fragments migrated as a single species on the non-reducing gel, demonstrating proper disulfide bond formation. Similar quantitative activation by thrombin digest was achieved for 150 kDa BoNT/A WT and WFY mutant, respectively (Supplementary Figure 4b). Accordingly, disulfide bridge formation between LC and H_N was observed under non-reducing conditions in SDS-PAGE. We believe the slight peak shift seen in Fig. 4a (retention volume 12.80 mL for WFY vs. 13.01 mL for BoNT/A WT = 0.88% of the column volume) is within the error range of the method. As suggested, we conducted structural investigation on BoNT/Ai-WFY through cryo-EM analysis and the result confirmed its structural integrity. Please see our response to Reviewer-1 for details.

5. The BoNT/A-WFY exhibits only ~40% of the wild-type activity in the MPN assay, whereas it shows more robust activity in the cleavage assay using neuronal cell culture. Please clarify this discrepancy. In addition, while the authors generally refer to BoNT/A, Supplementary Figure 4a specifies "engineered BoNT/A," which contains a short peptide including a thrombin site for additional cleavage. This should be described in the main text, along with an explanation of why this mutant protein was used.

Response:

The two functional assays we used here are very different. The MPN assay was performed within 1-3 hours under constant presence of 1-10 pM BoNT with 1 Hz electrical stimulation of exo-/endocytosis accelerating uptake of BoNT, using time to half-maximal muscle paralysis as a readout. In contrast, the P19 neurons were incubated with 0.3-300 pM BoNTs without stimulation for 2 h and then free BoNT was washed away. Subsequently, BoNT action was allowed for another 2, 6, 10 and 22 h to directly measure the intracellular SNAP-25 cleavage. It has been previously shown by Keller et al. (Biochemistry 2004) that evoked level of neurotransmitter release and SNAP-25 cleavage in mouse spinal cord cultures require different amounts of BoNT/A (IC_{50} 40 pM vs 589 pM). However, differences in IC_{50} can be overcome by different incubation times and/or absence of stimulation. In MPN assay, measurement of muscle

paralysis corresponds to neurotransmitter release whereas in P19 neuronal cell-based assay SNAP-25 cleavage was monitored in a much longer time-frame. The 1 Hz electrical stimulation used in the MPN assay drastically accelerates and increases the uptake of BoNT into the motor neurons allowing to measure the potency of minute BoNT concentrations, but may also cover the differences on the speed of LC translocation. In contrast, the absence of stimulation and the much longer time-frame in the P19N assay allows to dissect different speeds of LC translocation. Our newly determined structure of BoNT/Ai-WFY showed that it displays an increased structural flexibility due to loosening of the belt, which likely affects its overall stability and certain biochemical properties. These differences may contribute to the slightly different potency observed in the MPN assay, where toxins were applied to the organ bath chamber under constant physical mixing. We expect that future studies are needed to fully realize the therapeutic potential of the fast-acting BoNT/A variants, focusing on optimizing the designs following the same strategy, refining formulation, evaluating different dosing protocols, and conducting additional functional characterizations.

Biologically active BoNTs need to be proteolytically cleaved in the surface exposed linker connecting LC and H_N between the LC C-terminal Cys (C430 in BoNT/A) and HC N-terminal Cys (C454 in BoNT/A). Since e.g. trypsin causes also offsite cleavage leading to deactivation of BoNT/A, we previously demonstrated specific and highly efficient proteolytic activation of recombinantly expressed BoNT/A-F by inserting a thrombin recognition sequence into the LC-H_N-linker (Weisemann et al. Toxins 2015). Accordingly, an identical short peptide containing a thrombin cleavage site is introduced between the LC and HC of BoNT/A and BoNT/A-WFY. Both proteins were expressed in *E. coli*, isolated, subsequently activated by thrombin and purified further by SEC. This method has been well reported in the literature (ref. Weisemann et al. Toxins 2015, Lam et al. 2018, Liu et al. 2023), and we clarified this in the method section.

6. The authors conclude that the 2G11 antibody, which recognizes the belt-buckle, neutralizes BoNT/A by inhibiting the conformational change induced at acidic pH. However, since the HC domain moves flexibly relative to the LC-HN in the cryo-EM structure, the antibody does not appear to inhibit HC movement. Could the authors provide experimental evidence demonstrating that the antibody blocks the conformational transition?

Response:

The reviewer is correct that 2G11 doesn’t inhibit the movement of H_C in BoNT/A. Instead, 2G11 stabilizes the structural integrity of the LC and belt-buckle in BoNT/A, preventing conformational changes in this area that are needed for LC translocation. While this finding is evidenced in the cryo-EM structure, we haven’t been able to demonstrate this by biophysical and biochemical methods despite extensive efforts. For unknown reason, 2G11 has unusual strong affinity for the two dyes we use for the thermal shift assay and the ANS binding assay, which prevents us from examining the dye binding of BoNT/A in the presence of 2G11. Nevertheless, in our earlier studies (ref. 36), we provided experimental evidence demonstrating that VHH JLE-E9 binding stabilizes the structure of LC-H_N/E and inhibits its conformational changes induced by acidic pH. Since this VHH and 2G11 bind to homologous sites on BoNT/E and BoNT/A, respectively, it’s reasonable to believe that they share similar neutralizing mechanisms.

Minor

1. The authors conclude that the belt of BoNT/E has greater flexibility than that of BoNT/A, based on comparisons with previously reported crystal structures. It would be valuable for the authors to also discuss the reported cryo-EM structures of BoNT/E and BoNT/A (bound to SV2B), which may provide further insights into belt flexibility.

Response:

The cryo-EM structures of BoNT/E and BoNT/A were discussed in this manuscript (refs. 26, 29). Please also see our response to Reviewer-2, comment #3.

2. Please clarify how the percentage of cleavage activity was calculated from the Western blots in the P19 SNAP-25 assay. In particular, it would be helpful to specify the quantification workflow (band selection for full-length vs. cleaved species, background subtraction, and normalization to a loading control or total protein), confirm that signals were within the linear range, and provide the exact formula used.

Response:

The SNARE protein VAMP-2 served as control protein and was detected by the mouse monoclonal anti-VAMP-2 antibody clone 69.1. Intact SNAP-25 and its N-terminal cleavage product AA1-197 were simultaneously detected by the mouse monoclonal anti-SNAP-25 antibody clone 71.2 recognizing an epitope present in both entities. ECL signals were detected by the INTAS ECL ChemoStar system using the overexposure feature to maintain signal intensities in linear range. Band signal intensities on each Western blot were quantified using ImageJ and defining regions of identical areas to each band and a randomly selected region as background signal. Signal intensities of background region was subtracted from signal intensities of all other regions of the same Western blot. SNAP25 cleavage [%] was calculated as: [SNAP25 AA1-197] / ([SNAP25 AA1-197] + [SNAP25 AA1-206]) x 100.

We added these details to the Method section as: “Intact and cleaved SNAP-25 (AA1-197) were detected by the mouse monoclonal anti-SNAP-25 antibody clone 71.2 (#111111; Synaptic Systems GmbH, Göttingen, Germany) recognizing an epitope present in both entities while VAMP-2 serving as control protein was detected by the mouse monoclonal anti-VAMP-2 antibody clone 69.1 (#104211; Synaptic Systems GmbH, Göttingen, Germany). ECL signals were detected by the INTAS ECL ChemoStar system using the overexposure feature to maintain signal intensities in linear range. SNAP25 signal intensities were quantified by ImageJ software package and background subtracted. SNAP25 cleavage [%] was calculated as: [SNAP25 AA1-197] / ([SNAP25 AA1-197] + [SNAP25 AA1-206]) x 100. Results of three independent experiments were analyzed by GraphPad Prism 10 software.”

3. Line 89, “corresponding to the N-terminal segment of the HC…” This sentence should be revised for clarity.

Response:

We modified this sentence as the following: “The belt is a long, largely unstructured loop corresponding to the N-terminal segment of the HC immediately downstream of the cleavage site. After cleavage, the N-terminus of the belt remains covalently linked to the C-terminal end of the LC through a disulfide bond”.

4. Line 438, please confirm whether “magnification of 29K” is correct?

Response: Yes, we confirmed the magnification is 29 K.

5. Could the authors clarify how the sequence of 2G11 was determined? If possible, providing the amino acid sequence would enhance the manuscript’s completeness.

Response:

Studies of 2G11 have been extensively reported by the Marks lab (refs. 32-35). Its amino acid sequence is now included in Supplementary Table 2.

6. Lines 266–275: This section seems somewhat confusing, and the manuscript may read more clearly if it is omitted.

Response:

This section is to address the potential safety concern since 2G11 can no longer recognize BoNT/A-WFY. We confirmed that BoNT/A-WFY can be neutralized by the antitoxin mixture under clinical trial and the licensed polyclonal antitoxin (BAT). To improve the readability, we moved this section to the end of the Results under an independent heading, “BoNT/A-WFY can be neutralized by the available antitoxins”.

7. Line 761: The description of the black arrows is unclear. It may be more informative to indicate the specific residues contributing to the interaction instead.

Response: We modified Fig. 1e as suggested, which now shows the specific residues contributing to the interaction.

8. Figure 2d, Does “BoNT/E (open)” refer to “BoNT/E (M-PTC form)”?

Response:

Yes. The “BoNT/E (open)” conformation shown in Fig. 2d was prepared using the structure of the LC-H_N fragment after focused refinement, which was resolved at a higher resolution compared to the Class 1 (M-PTC form) structure shown in Fig. 2a-b.

9. Related to Figure 3f, the melting curve must be provided in the Supplementary.

Response:

We have included representative melting curves as well as all the raw data in the Source Data associated with Figure 3f.

10. In Supplementary Figure 2 and 5, higher resolution figures must be provided. Especially, the micrographs are of poor quality.

Response: Figures are optimized as suggested.

Response to Reviewer 3

While Both of BoNT/A and BoNT/E. employ the synaptic vesicle glycoprotein 2 (SV2) as the receptor, there are three paralogs of SV2s (A,B,C). It is my understanding that the respective affinity by BoNtA and E differ with the former showing the highest affinity for SV2C and the later employing, SV2A,B. and not C. I am not clear on the differential distribution of the paralogs in the cholinergic terminal (e.g. relevant to the hemidiaphram). Please address the speculation that the differential onset of block might reflect that all three paralogs are in the cholinergic terminal and the onset reflects a more a more rapid uptake cleavage is not due to the role of SV2A/B for BoNtE and only on SV2C for BoNtA.

Response:

Based on the literature, all three SV2 paralogs are expressed on motoneurons. Since the relative abundance of their expression on cholinergic terminals is not clear, we don't know how this will affect the onset for BoNT/A and BoNT/E. However, based on our earlier studies and literature, the binding affinity of BoNT/E to SV2A/2B is weaker than that of BoNT/A, suggesting that SV2 binding affinity is unlikely contributing to the faster onset of BoNT/E.

It is clear to me that the time course of unfolding of the BoNT construct is regulated by the tightness of the belt and that where the target effect is local (as in delivery to the cutaneous cholinergic terminal), changing the belt tightness can enhance the unfolding revealing the active form. Here the time course. largely depends upon the time of uptake and then upon time of onset of the consequence of the cleavage.

There is, however, considerable evidence that many effects of several isoforms of BoNT may be mediated by its uptake in the terminals and transport to the cell bodies. Such BoNT transport has been well documented. Models focusing on the time to onset of effects upon pain behavior (PMID: 39196607; PMID: 38452215.; PMID: 25958249) and the earlier effects upon plasma (PMID: 24333775 are consistent with the cleavage of SNAREs in the terminals and then the DRG.

In that I regard, I note that BoNT/A and BoNT/E has been reported to be. internalized in non-acidic axonal carriers and undergo fast axonal retrograde transport in motor neurons. BoNT A reportedly at rates of 0.8 µm/s (PMID: 23300443). I do not know such data on BoNT E transport velocities and while it is not likely to be that much more more rapidly transported than the BoNtA, it still reflects upon the potential role of transport velocity n defining the time of effect onset.

Would you care to speculate on the role of belt tightness on such ortho /antidromic transport and perhaps note that some clinically relevant delays may reflect time to affect the central terminals.

Response:

We agree that the body of literature investigating and demonstrating central effects especially by BoNT/A is growing. Such central effects upon peripheral injection can only be explained by retrograde axonal transport which was identified by seminal work of Caleo et al. (Antonucci et al. 2008 and following papers). Along that line Restani et al. 2012 demonstrated that peripheral

effects of BoNT/A occur in vivo in rats within 24 h whereas SNAP-25 cleavage in the respective motoneuron nuclei was just detected after 72 h supposedly due to the retrograde transport time of 48 h of BoNT/A. Several studies showed in in vitro neuronal cell-based assays that peripheral/local action at the NMJ occurs at picomolar concentrations within 1-4 h for BoNT/E and 8-24 h for BoNT/A confirming the quick onset of action of BoNT/E and delayed onset of BoNT/A in vivo. Restani et al. 2012 also incubated isolated rat spinal cord motor neurons with 30 nM of fluorescently-labelled BoNT/A and BoNT/E, respectively, for 30 min at 37°C at resting conditions. Subsequently, they observed retrograde axonal transport of fluorescently labelled BoNT/A and BoNT/E with equal speed distribution profile suggesting that BoNT/A and E reach the facial nuclei at similar time and excluding a role of the tightness of the belt for transport speed. At the soma similar to the periphery, central effects could occur for BoNT/E in 1-2 hours and for BoNT/A in 4-8 h. Considering 48 h of transport time for each serotype, an onset of central action of BoNT/A delayed by 3-6 h vs BoNT/E seems to be negligible and insufficient to explain clinically observed delayed central effects upon intoxication by BoNT/E or BoNT/A.

Reviewer #1 (Remarks to the Author):

I think the submitted manuscript is greatly improved, but it requires additional work. Although the authors have adequately addressed most of the comments, the new structure of BoNT/Ai-WFY is deeply problematic. There is basically no structure - just an overfitted 3D volume with essentially no protein-like features. The supplied density map is a collection of loosely connected hollow volumes and there is no way this can be published in the current form. What the authors have at the moment is not a cryo-EM structure at 5Å resolution but a hallucination.

Because of this, the manuscript requires careful reevaluation. The new structural data on the mutant must be presented in a different way, and I think it is ok to realise that this mutant may not produce a high resolution structure. If the cryo-EM imaging of WFY is to be kept in the manuscript, the corresponding cryo-EM analysis must be at least conclusive.

The authors might want to consider continuing 3D classification to try and extract a better 3D volume from their current one (301,287 particles is a large dataset). Whether this will produce any results is currently hard to judge, but it's worth trying.

Response: We reprocessed the dataset following reviewer's suggestions. Because of the increased structural flexibility introduced by the WFY mutation, the particles were of relatively low quality, which significantly hindered both 2D alignment and 3D reconstruction. We performed additional local refinement and 3D classification; however, the resulting density maps remained of low quality and did not reveal reliable high-resolution structural features. We further screened the dataset to ~20k particles and obtained a similar result, indicating that the limitation was due to sample heterogeneity and conformational flexibility (new Supplementary Fig. S6). The related discussion in the main text has been revised accordingly. We also performed negative-stain EM to further assess the overall structural integrity of BoNT/Ai-WFY, as described below.

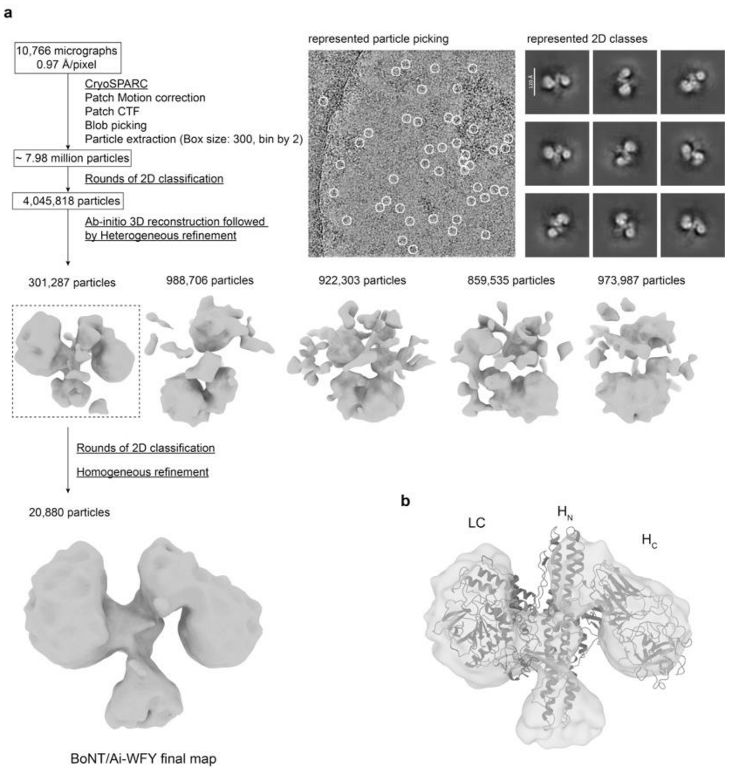

Supplementary Figure 6. The workflow of cryo-EM analysis of BoNT/Ai-WFY. a. The workflow of cryo-EM data processing of BoNT/Ai-WFY. Examples of a cryo-EM micrograph and 2D classes were shown in the right panels. **b**. The crystal structure of wild-type BoNT/A (PDB: 3BTA) is fitted into the BoNT/Ai-WFY density map to illustrate the overall domain arrangement.

The local resolution-coloured map in its current form is basically meaningless (Supplementary Figure 5), because the volume that was produced by non-uniform refinement lacks any clear features.

Response: We agree with the reviewer and have removed this part accordingly.

The authors wrote "The overall structure of BoNT/Ai-WFY is almost identical to the crystal structure of WT BoNT/A (PDB: 3BTA) (new Supplementary Fig. 5-6, next page)." in the rebuttal. Even with the most charitable approach to the available "3D reconstruction" of BoNT/Ai-WFY, the fit of the 3BTA is very poor. The HN domain matches to a region where there is no density. The extra bit of density in the bottom of the blobby map in Supplementary Figure 6 does not match the HN domain (or any other domain of the toxin). Regardless of all this, it is not worth spending any time analysing the fit of an X-ray structure to an overfitted 3D volume that does not correspond to the protein of interest.

Given the current lack of any conclusive results on cryo-EM analysis of the WFY mutant, that whole part of the manuscript needs to be reworked and supplementary figures 5-6 need to be modified accordingly. It is possible that there will not be a structure (even at low resolution) despite the best efforts of the authors. They should consider removing this part from the manuscript, should they reach this conclusion.

Response: We have substantially revised this section and removed the previous overinterpretation of the cryo-EM reconstruction as suggested. To further assess the overall structural integrity of BoNT/Ai-WFY, we performed negative-stain EM on BoNT/Ai-WT and BoNT/Ai-WFY, followed by 2D classification.

Representative class averages from both samples showed similar particle shapes and domain organization, closely resembling the canonical architecture of wild-type BoNT/A (PDB: 3BTA). In these averages, the LC, H_N and H_C domains could be readily recognized based on the reference structure (new Supplementary Fig. 5a-b). No obvious large-scale conformational differences were observed between BoNT/Ai-WT and BoNT/Ai-WFY at the negative-stain level, suggesting that the WFY mutations do not substantially perturb the overall architecture of the toxin.

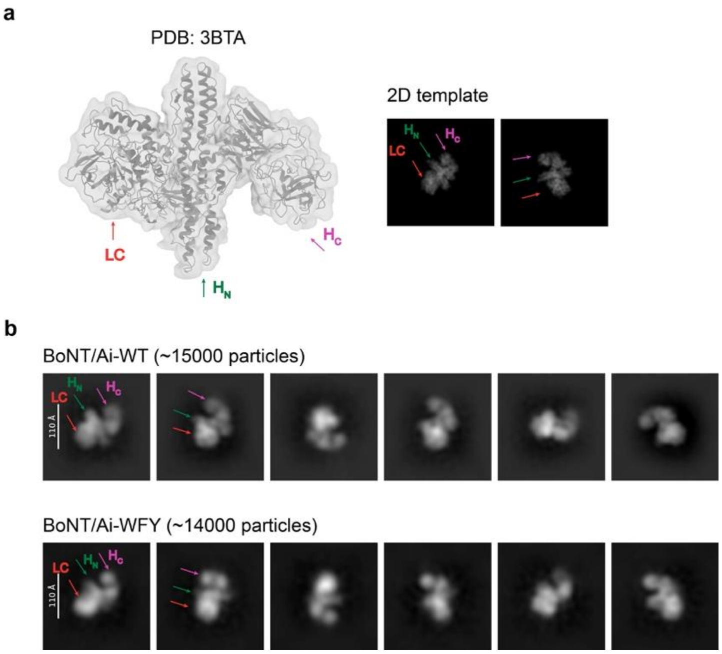

Supplementary Figure 5. Negative-stain EM analysis of BoNT/Ai-WT and BoNT/Ai-WFY. a. Structure of wild-type BoNT/A (PDB: 3BTA) highlighting the three major domains: light chain (LC, red), translocation domain (H_N, green), and receptor-binding domain (H_C, magenta). Representative 2D template views generated by CryoSPARC are shown on the right, with arrows indicating the approximate positions of each domain. **b**. Representative 2D class averages of BoNT/Ai-WT and BoNT/Ai-WFY obtained from negative-stain EM. Selected class averages are shown, with arrows marking the approximate positions of LC, H_N, and H_C.

The revised text is as follows:

"We next examined whether weakening the belt-buckle with the WFY mutations might affect the structure and function of BoNT/A. To this end, we recombinantly produced the wild-type and WFY variant of BoNT/A holotoxin in E. coli. Each construct contains a thrombin recognition site engineered into the flexible linker connecting LC and H_N, enabling controlled activation without disturbing the structure or activity of BoNT/A[23,44,45]. BoNT/A-WFY could be purified to homogeneity and activated in a manner comparable to WT BoNT/A (Fig. 4a and Supplementary Fig. 4b). We also produced the inactivated BoNT/Ai-WFY for structural characterization.

To assess whether weakening the belt affects the overall architecture of BoNT/A, we performed negative-stain EM on BoNT/Ai-WT and BoNT/Ai-WFY, followed by 2D classification. Representative class

averages from both samples showed similar particle shapes and domain organization, closely resembling the canonical architecture of wild-type BoNT/A (PDB: 3BTA)[27]. In these averages, the LC, H_N and H_C domains could be readily recognized based on the reference structure (Supplementary Fig. 5a-b). No obvious large-scale conformational differences were observed between BoNT/Ai-WT and BoNT/Ai-WFY at the negative-stain level, suggesting that the WFY mutations do not substantially perturb the overall architecture of the toxin. However, our attempts to determine a high-resolution structure of BoNT/Ai-WFY by cryo-EM were unsuccessful. Despite extensive image processing, only a low-resolution density map was obtained, indicating increased structural flexibility, likely arising from loosening of the belt region (Supplementary Fig. 6a-b).”

<u>Reviewer #2 (Remarks to the Author):</u>

I appreciate the authors’ faithful efforts in addressing the previous comments. The newly presented cryo-EM structure of BoNT/Ai-WFY is reported at approximately 5 Å resolution; however, even secondary structure elements do not appear to be well resolved (Supplementary Figure 6). While I understand the authors’ interpretation that this may be due to increased structural flexibility, the current cryo-EM map appears more indicative of overall structural destabilization rather than localized domain movement. At the very least, it is not entirely convincing to conclude that the overall structure is nearly identical to that of the crystal structure of the wild-type protein (Page 6). Could the authors clarify whether additional efforts were made to improve the map quality, such as local refinement or more extensive 3D classification? The authors report selecting approximately ~30 k particles as the best class from an initial ~400k particles. It would be helpful to know whether other classes were also evaluated and whether any of them yielded maps with improved resolution.

Response: We thank the reviewer for the thoughtful comments and careful evaluation of the BoNT/Ai-WFY cryo-EM data. We have substantially revised this section and removed the previous overinterpretation of the cryo-EM reconstruction as suggested. To further assess the overall structural integrity of BoNT/Ai-WFY, we performed negative-stain EM on both BoNT/Ai-WT and BoNT/Ai-WFY. Please see our responses to Reviewer-1 for details.

The BoNT/As used in the activity assays are mutant proteins introducing thrombin cleavage sites. It is unclear whether such mutants are commonly used for activity assays in this field. Although the authors added the explanation about this in the methods in the revised manuscript according to the reviewer's comment, this point should be clarified in the main text (page 6, around MPN assay), along with an explanation of how the introduced mutations may affect the activity and interpretation of the results.

Response: This method is well established in the field. As part of the EU-funded EQuATox project, a recombinant BoNT/A was produced using the same thrombin activation strategy to serve as a certified reference material. This recombinant BoNT/A was extensively evaluated across multiple laboratories within the EQuATox consortium and exhibited toxicity compared to that of commercially available native BoNT/A purified from *C. botulinum* (ref. 45). We have included this information in the main text along with three related references (ref. 23, 44, 45).

I still have concerns regarding the reported magnification of 29k×. The dataset for BoNT/Ai-2G11 was collected at 105k× magnification with a pixel size of 0.8266 Å, whereas the dataset for either LC-HN/E or

BoNT/Ei was collected at 29k× with a pixel size of 0.788 Å. As both datasets were acquired using the same microscope at the same facility, the smaller pixel size for the latter case would typically correspond to a higher nominal magnification than 105,000× of the former case. Could the authors please verify whether the reported magnification and pixel size values are correct?

Response: We thank the reviewer for this careful observation. We confirmed the reported values are correct. The apparent discrepancy arises because the displayed nominal magnification is microscope-specific and depends on the detector configuration along the column. In this case, the two datasets were collected on different Titan Krios instruments at PNCC with different setups (Krios #1 for BoNT/Ei, Krios #4 for BoNT/Ai-2G11): Krios #1 did not have an energy filter installed, whereas Krios #4 was equipped with a Gatan BioContinuum energy filter. As a result, the specimen-to-detector distance was shorter on Krios #1, which changes the relationship between nominal magnification and calibrated pixel size. Therefore, although the BoNT/Ei dataset was collected at 29k× with a pixel size of 0.788 Å and the BoNT/Ai-2G11 dataset was collected at 105k× with a pixel size of 0.8266 Å, these values are correct and are not directly comparable by nominal magnification alone.

I found several typos. For example, "Thermal Fisher Vitrobot M" in the last sentence on Page 12 should be "Thermofisher Vitrobot Mark IV". Please proofread the entire manuscript carefully.

Response: We have carefully proofread the manuscript and corrected typos throughout.

Reviewer #3 (Remarks to the Author):

The response to my review is appropriate and adequate. My view of the potential contribution of transport to the to the time of onset issue while not relevant to the results of the in vitro/ex vivo results characterizing the role of the belt are potentially relevant to the issue of time of onset for event that almost certainly reflect upon a central effect of BoNTs delivered to the peripheral terminal (e.g as in the use in managing pain of a neuropathic origin and in migraine.

Tony L. Yaksh

Response: Thanks.

Point-by-point response

Reviewer #1 (Remarks to the Author):

I am overall satisfied with the revised manuscript. I think the authors will have to accept the fact that the WFY mutant is substantially perturbed, even if the negative stain suggests that similar shapes can be observed for this mutant as for the wild-type. I am not sure the wording they choose in the description of the negative stain results is optimal, but I leave it to the authors to decide how to deal with this.

It is clear that the construct is badly affected, so a statement like "No obvious large-scale conformational differences were observed between BoNT/Ai-WT and BoNT/Ai-WFY at the negative-stain level, suggesting that the WFY mutations do not substantially perturb the overall architecture of the toxin" can be misread / misinterpreted by the reader, even if a disclaimer follows in the following sentences. In my opinion it would be safer to avoid the words "conformational" (the conformation of WFY clearly is very much affected, and the negative stain data hardly substitute cryo-EM analysis in this case) and "architecture" (this word is not defined here, and given the clear disruption of the structure this is just confusing). The 2D classes of the WFY in negative stain are similar, but not the same as those of the wild-type, so the statement about the large-scale conformational differences is probably not quite right.

There must be a more straightforward way to state that the WFY mutant structure is perturbed / destabilized, even though negative staining of the sample indicates that the gross features of the particles appear to be preserved.

Response:

We have revised the discussion related to the structure of BoNT/Ai-WFY, and the revised text now reads as follows:

"To assess whether weakening the belt affects the overall architecture of BoNT/A, we performed negative-stain EM on BoNT/Ai-WT and BoNT/Ai-WFY, followed by 2D classification. Representative class averages from both samples showed similar particle shapes and domain organization, broadly resembling the canonical structure of wild-type BoNT/A (PDB: 3BTA)[27]. In these averages, the LC, H_N and H_C domains could be readily recognized based on the reference structure (Supplementary Fig. 5a-b). While some structural differences may exist in BoNT/Ai-WFY, the gross features of the particles appear to be preserved at the negative-stain level. Our attempts to determine a high-resolution structure of BoNT/Ai-WFY by cryo-EM were unsuccessful, and only a low-resolution density map was obtained (Supplementary Fig. 6a-b). These findings are consistent with increased structural flexibility, likely arising from loosening of the belt region."
